# Sequence analysis and decoding with extra low-quality reads for DNA data storage

**DOI:** 10.1093/bioinformatics/btaf335

**Published:** 2025-06-10

**Authors:** Jiyeon Park, Ha Hyeon Jeon, Jeong Wook Lee, Hosung Park

**Affiliations:** Department of Intelligent Electronics and Computer Engineering, Chonnam National University, Gwangju 61186, South Korea; Department of Chemical Engineering, POSTECH, Pohang 37673, South Korea; Department of Chemical Engineering, POSTECH, Pohang 37673, South Korea; Department of Intelligent Electronics and Computer Engineering, Chonnam National University, Gwangju 61186, South Korea

## Abstract

**Motivation:**

Error detection/correction codes play an important role to reduce writing and/or reading costs in DNA data storage. Sequence analysis algorithms also make a crucial effect on error correction but have been executed independently from the decoding of error correction codes. In conventional sequence analysis, low-quality reads are usually discarded. For DNA data storage, low-quality reads can be constructively used to sequence analysis with the assistance of error detection/correction codes.

**Results:**

We obtained the low-quality reads which failed to pass the chastity filter in Illumina NGS sequencing. We confirmed the effectiveness of the extra low-quality reads by providing error statistics and performing decoding with them. We proposed a sequence clustering algorithm for various-length reads and a consensus algorithm based on probabilistic majority and error detection to efficiently exploit the extra reads. The proposed methods reduced the reading cost by 6.83% on average and up to 19.67% while maintaining the writing cost.

**Availability and implementation:**

https://github.com/PParkJy/SAD-DNAstorage (10.5281/zenodo.15571858).

## 1 Introduction

With the growth of IT services and smart devices, the amount of data to be stored is rapidly increasing. Most of the data is cold data that is rarely used for now but must be stored for a long time to meet future demand (Shobana Balakrishnan *et al.* 2014). Many companies have data centers to store these data, but storage mediums currently used in data center have limitations such as power consumption and durability (Bornholt *et al.* 2016). Hence, a new storage medium is required, and deoxyribonucleic acid (DNA) is being actively researched as a strong candidate. DNA is suitable for long-term storage of massive data because it provides long lifetimes, high data density, and low power consumption (Church *et al.* 2012, Bornholt *et al.* 2017).

The process of DNA data storage can generally be classified into synthesis, polymerase chain reaction (PCR), and sequencing. In DNA data storage, digital data are stored in short DNA molecules called oligonucleotides, shortly oligos. The nucleotides in DNA have four bases called adenine (A), cytosine (C), guanine (G), and thymine (T), and digital data bits are mapped to DNA bases to generate oligo sequences. Then, artificial oligo molecules are produced following the oligo sequences through a synthesis process. PCR is a technique used to amplify the number of oligo molecules. The oligo molecules are sequenced to generate sequence reads. The stored digital data are retrieved by converting the bases of sequence reads into bits. However, this may not be a perfect data retrieval because the biochemical process of DNA data storage cannot be perfectly controlled. Substitution, deletion, and insertion errors generally occur during the writing and reading of data (Heckel *et al.* 2019).

To ensure the reliability against errors, various studies have been conducted to apply error detection/correction codes. Encoding process which adds redundancy to the original data is performed before synthesis, and decoding which corrects the errors in the sequence reads to recover the original data is carried out after sequencing. In many cases, a short code is applied to each oligo sequence to detect errors within the sequence and a long code is applied across all oligo sequences to recover sequence erasures incurred by dropouts or forced by error detection in the oligo sequence. The former and the latter codes are called inner and outer codes, respectively. For example, in Grass *et al.* (2015), Reed–Solomon (RS) codes were used for both inner and outer codes to completely recover an 83 KB text file. In Organick *et al.* (2018), the same code structure was employed for random access and recovered over 200 MB of data. Also, in Matange *et al.* (2021), RS codes were used as both inner and outer codes for error detection and correction. In Erlich and Zielinski (2017) and Anavy *et al.* (2019), Luby-Transform (LT) code was adopted as an outer code, a kind of fountain code for erasure recovery and RS code was adopted as an inner code for error detection. In Blawat *et al.* (2016), the authors utilized outer RS code and inner cyclic redundancy check (CRC), and BCH code for oligo address protection. In Chandak *et al.* (2020), the authors used outer RS code and inner convolutional code along with CRC error detection. In Press *et al.* (2020), the authors also used outer RS code and hash-based inner code.

There have been also many researches on the design of error correction codes for DNA storage. To address challenging insertion and deletion (indel) errors, single-indel correction codes were proposed in Cai *et al.* (2022) and Wu and Zhang (2024). In Con *et al.* (2024), a 2D RS code was designed to correct multiple indels. To account for the unordered nature of DNA storage, index-based codes were constructed in Lenz *et al.* (2020). In Weinberger (2022), error probabilities of coded-index schemes were analysed, and a robust indexing based on RS code was proposed in Sima *et al.* (2025). In Bar-Lev *et al.* (2025), the authors proved that maximum distance separable codes are optimal for reducing the expected number of sequenced reads under noiseless or uniformly random channels. To suppress errors, various works (Cai *et al.* 2021, He *et al.* 2025) have focused on the code designs that satisfy constraints such as GC-content and homopolymer-run.

Using these codes improves the tradeoff between writing and reading costs of DNA storage (Heckel *et al.* 2017, Park *et al.* 2023). One approach to achieve a good tradeoff is to collect as many error-free sequences as possible and input them to the decoder of the outer code because the decoder can easily recover a small number of oligo erasures. To obtain error-free sequences, existing studies have applied the built-in sequence analysis of the next generation sequencing (NGS), followed by secondary analysis such as sequence clustering and alignment (Jeong *et al.* 2021). Despite the capabilities of inner codes, conventional sequence analysis methods have been performed independent of error detection and decoding. Incorporating error correction codes into sequence analysis could make it possible to obtain more error-free sequences.

Generally, a number of reads are discarded during the sequence analysis due to unreliable basecalling, low-quality score, abnormal length, and detected error. Illumina’s chastity filter discards low-quality reads during basecalling process. By following Illumina’s naming (Illumina 2016), we will call the reads passing the chastity filter with an identified index pattern ‘pass filter (PF) reads’ and the other reads ‘non-pass filter (NPF) reads’. In most previous researches, DNA storage used only PF reads to recover the original data. However, not all the NPF reads are seen severely contaminated and a constructive usage of NPF reads could help generate more error-free sequences especially when considering the post-filtering from the error detection of inner codes. In addition, abnormal-length reads and/or error-detected reads also have the potential to generate error-free sequences through well-designed sequence analysis with error correction codes, if they were not discarded.

In this article, we propose an elaborate usage of low-quality reads that were previously discarded in conventional DNA data storage for improved error correction from sequence analysis and decoding. To demonstrate the characteristics of NPF reads, we analysed the error statistics of NPF reads compared to those of PF reads. We also designed a step-by-step sequence analysis workflow to effectively utilize the low-quality reads. It includes a sequence clustering algorithm to group various-length reads around error-detected original-length reads based on edit distance (Levenshtein *et al.* 1966) and a consensus algorithm to iteratively determine a final sequence per cluster based on probabilistic bit majority voting and error detection. Our method can be applied to a wide range of DNA data storage systems with the structure of inner and outer codes since it does not rely on specific error correcting codes. Compared to the conventional method (Erlich and Zielinski 2017), the proposed method resulted in up to 10.28% reduction in reading cost while maintaining writing cost.

## 2 Materials and methods

### 2.1 Encoding, synthesis, PCR, and sequencing

**Figure 1.**
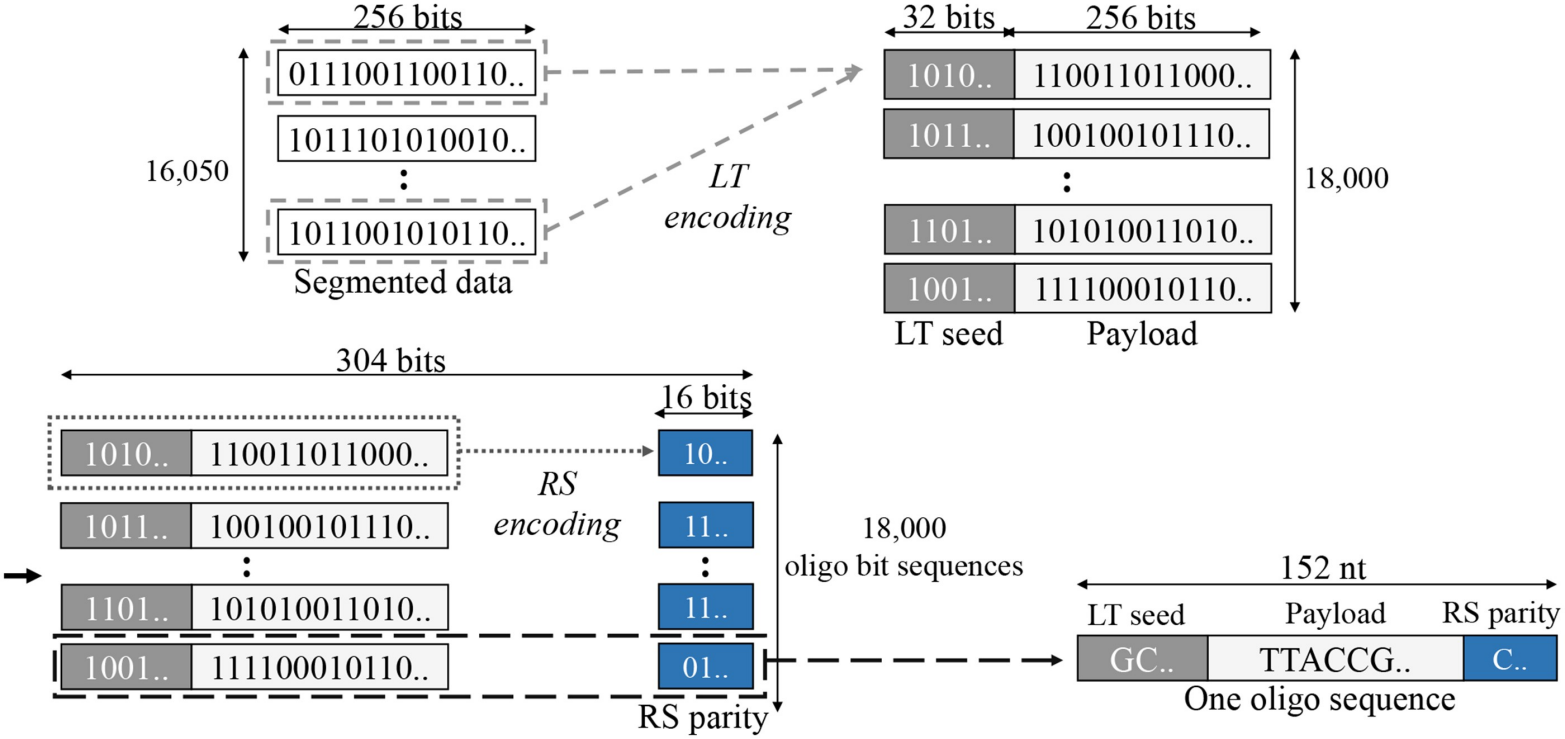
Encoding with LT and RS codes for DNA oligo sequences.

We encoded a 513.6 KB of image data into 18 000 oligo sequences of 152 nucleotides (nt) as shown in Fig. 1. We followed the method of Erlich and Zielinski (2017) to ease the generation of constrained sequences suppressing errors (Schwarz and Freisleben 2024) while maintaining high information density (Ping *et al.* 2022).

The image bits were divided into 16 050 segments of 256 bits each. An LT code was used as the outer code to generate the 18 000 payloads of 256 bits by linearly combining the segments, which corresponds to adding 12% redundancy by the LT code. A 32-bit LT seed was prepended to each payload for identification. To protect the LT seed and payload at once, we applied a (38, 36) RS code over the Galois field GF($2^{8}$) as the inner code to generate 16-bit RS parity bits appended to each payload. As a result, one oligo sequence consists of 304 bits mapped to bases according to the rule: A = 00, C = 01, G = 10, T = 11. When using the LT code, we ensured the 18 000 payloads satisfy the constraints of homopolymer-run length less than four and a GC-content in the range of 45%–55%.

By attaching 26 nt and 21 nt Illumina adapters to the head and tail of the oligo sequence, respectively, 18 000 oligos have length 199 nt. A 300 ng oligo pool was synthesized by Twist Bioscience. PCR amplification was performed with RP1 and RPI1 primers through thermocycling cycles. The PhiX Control v3 was mixed by 30% to form the library and sequencing was carried out with Illumina MiSeq Reagent Kit v3 (600-cycle). In total, 308 cycles were run for paired-end sequencing: the first 151 cycles for the forward direction, followed by six cycles for indexing, and the last 151 cycles for the reverse direction. The adapter and the PCR experimental details are described in Supplementary Material S1, available as supplementary data at *Bioinformatics* online.

### 2.2 Basecalling from raw sequencing data

The NPF sequence reads are not stored in any output files by default, so we exceptionally obtained raw data as a format of cif file by modifying Illumina MiSeq’s configuration settings. The cif files are generated for every cycle and each cif file includes the intensities of four channels for all clusters, which will be converted to both PF and NPF reads. We used the AYB basecaller of version 2.11 (Massingham and Goldman 2012) to generate all the sequence reads from the cif files. Illumina MiSeq outputs a format of filter files indicating whether to pass the chastity filter, so we used them to classify the sequence reads into PF and NPF. To the best of our knowledge, only PF reads have been used for sequence analysis and decoding in previous studies with Illumina sequencing because many errors are expected in NPF reads. In DNA storage, we can use error detection/correction codes for each oligo, so we can utilize the NPF reads to generate as many error-free sequences as possible when selectively used with tailored sequence analysis algorithms.

### 2.3 Sequence analysis and decoding

In this work, the RS code only detects errors in each oligo sequence to identify error-free sequences. The LT decoder runs the iterative erasure decoding with only the error-free sequences and tries to recover the oligo erasures which are not identified by the error-free sequences. With the fixed outer erasure decoding strategy, we proposed clustering and consensus algorithms incorporating the inner error detection which leverage the extra low-quality reads.

#### 2.3.1 Preprocessing

For accurate algorithms, we performed the following preprocessing steps on the sequence reads. We deleted the last base of each read to have length 150 because sequencing in the last cycle is known unstable. Some reads may include a part of the adapter pattern at the tail due to the pre-phasing of sequencing. We used fastp of version 0.20.1 (Chen *et al.* 2018) to trim these adapter patterns only when their lengths are greater than or equal to seven. After the trimming, PEAR of version 0.9.11 (Zhang *et al.* 2014) was used to merge the pair-end reads. The merged reads were allowed to have various lengths though the original oligo length is 152.

#### 2.3.2 Clustering, alignment, and consensus algorithms

**Figure 2.**
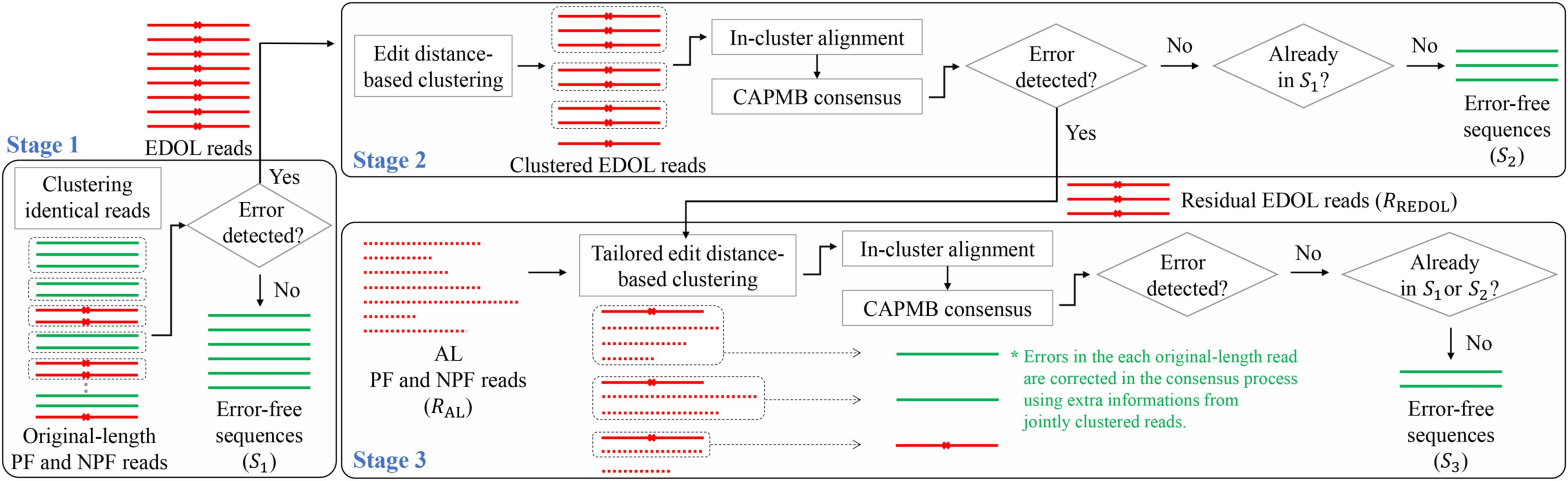
The proposed sequence analysis workflow. Reads that fail error detection at each stage move on to the next stage. Stage 1 uses existing sequence analysis methods using only original-length reads. Stage 2 focuses on correcting EDOL reads by edit distance-based clustering with the proposed CAPMB consensus algorithm. Stage 3 applies tailored edit distance-based clustering that ignores indel errors caused by differences in read lengths and additionally use AL reads with the CAPMB consensus to correct the EDOL reads. The error-free sequences generated in each stage are fed into the LT decoder sequentially.

Reads that retain their original length but have errors detected by the inner code will be referred to as ‘error-detected original-length (EDOL) reads’. Reads with abnormal length already contain errors, and these reads will be termed to as ‘abnormal-length (AL) reads’. Many sequence reads are error-free with the original length but there are also EDOL and AL reads. Especially, we allow to additionally use the NPF reads so the portion of erroneous reads can be higher than previous studies. Clustering, alignment, and consensus algorithms enable error correction because we have multiple called bases within a cluster for each base position and a consensus by majority vote becomes to have a lower error probability than a single basecall. The EDOL reads with few errors have potential to be corrected in combination with other reads. Though the AL reads themselves are obviously erroneous, they can also help generate error-free sequences when properly clustered with other reads. To improve data recovery rates for the given outer erasure decoding, we aim to maximally generate error-free sequences for the input to the decoder by designing a progressive three-stage workflow of clustering, alignment, and consensus algorithms as shown in Fig. 2. The proposed sequence analysis workflow deals with the EDOL and AL reads from both PF and NPF reads and incorporates the inner error detection to boost its error correction capability.

**Stage 1:** Stage 1 exactly follows the method in Erlich and Zielinski (2017) for the PF and NPF reads. Only identical PF and NPF reads with the original length are grouped into the same cluster. Then no multiple sequence alignment is required and the consensus is trivial. After that, all the consensus sequences are sorted by cluster size and go through error detection. The error-free sequences are denoted by $S_{1}$ and input to the outer decoder one by one.

**Stage 2:** In Stage 2, an edit distance-based clustering is performed for the EDOL reads which were not input to the decoder in Stage 1. After multiple sequence alignment for clusters, an inner code-aided consensus algorithm is executed to further generate error-free sequences. For the edit distance-based clustering, we used the sphere clustering of the starcode (Zorita *et al.* 2015). Starcode has an input parameter of the maximum edit distance $\tau_{e}$ and efficiently calculates whether the edit distance between two sequences is within $\tau_{e}$. Since a large $\tau_{e}$ can incur clustered reads originating from difference information sequences and a small $\tau_{e}$ can miss some useful reads originating from the same information sequence in the cluster, a proper setting of $\tau_{e}$ is important depending on the code structure and the error statistics of the DNA storage. Then, MUSCLE of version 5.0.1428 (Edgar 2022) was used for multiple sequence alignment for every cluster.

**Figure 3.**
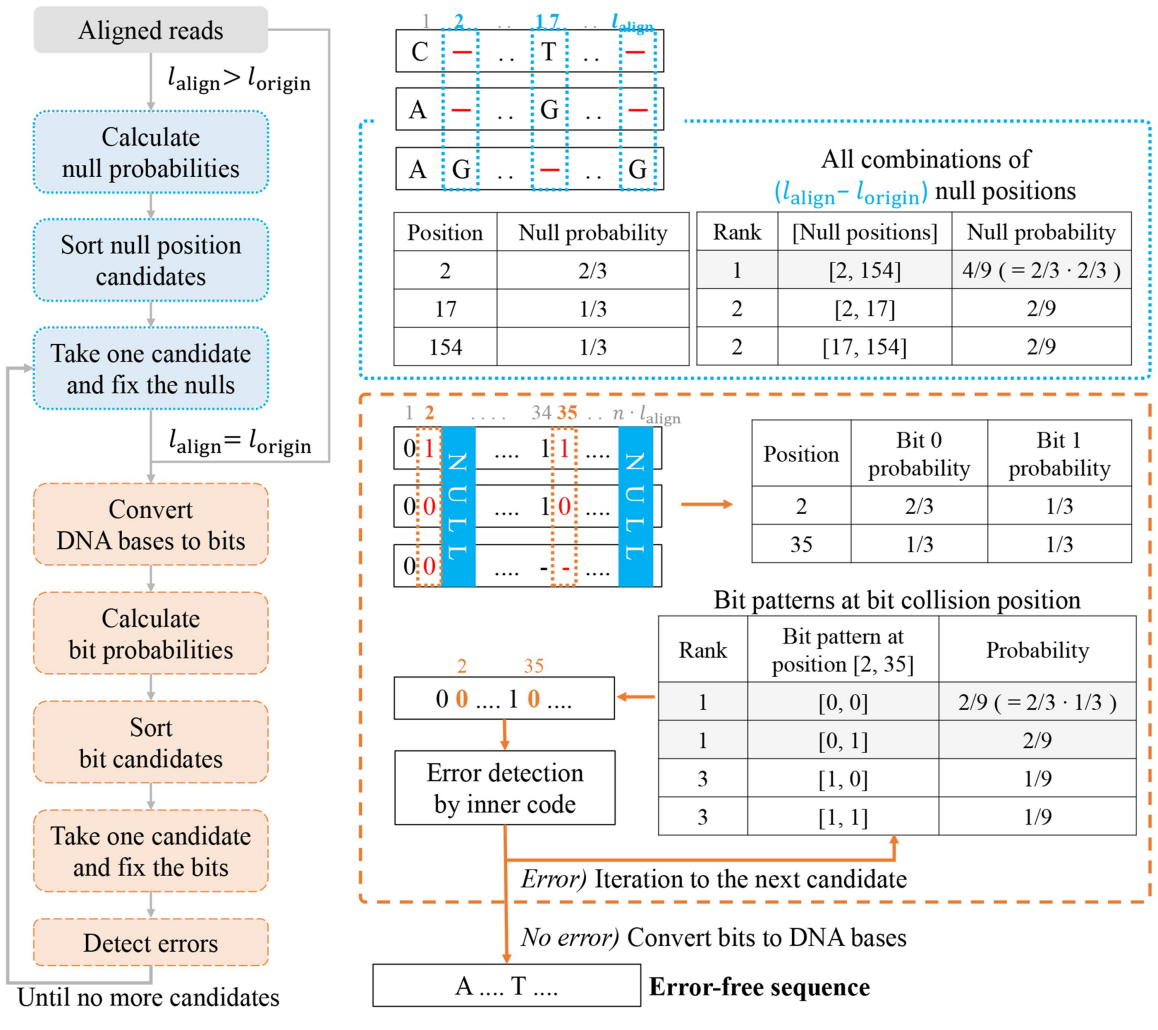
Proposed CAPMB consensus algorithm for Stages 2 and 3. In the case that the length of the aligned reads $l_{\text{align}}$ is longer than $l_{\text{origin}}$, ($l_{\text{align}}-l_{\text{origin}}$) nulls are selected to ensure that the length of the consensus sequence is $l_{\text{origin}}$. In the case that $l_{\text{align}}$ is equal to $l_{\text{origin}}$, the bases are converted into the bits. The number of bits mapped to each base *n* are depending on the encoding method. In our case, $l_{\text{origin}}$ is 152 and *n* is 2. We used the RS code for inner code but any error detection/correction code can be applied.

A common way to determine the consensus sequence for a cluster is to use the majority decision which selects the most frequent base at each position. If the cluster size is small and/or the clustered reads are much erroneous, the resulting consensus sequence would still include errors and be discarded by error detection. At this moment, we have powerful options to lead out more accurate consensus sequences by actively utilizing the function of inner codes. Instead of taking only one majority, we allow to consider multiple majorities from the highest frequency in each base position. Then, we can form a set of many candidates for a consensus sequence because there are usually multiple base positions which are not unanimous. Among the candidates, we try the error detection one by one from the most probable sequence and the first candidate passing the error detection becomes the output of this process. We call this a code-aided probabilistic majority-based (CAPMB) consensus algorithm (Fig. 3).

During alignment, some reads get to contain nulls denoted by ‘-’ character, which mean the absence of bases. Let $l_{\text{origin}}$ and $l_{\text{align}}$ denote the original and aligned read lengths, respectively. In case of $l_{\text{align}}>l_{\text{origin}}$, the aligned reads are additionally processed with a null position determination before the bit-level consensus. Among the aligned reads, we store all the base positions where nulls occur at least once and calculate the null probability per position based on frequency. Since error-free sequences should have $l_{\text{align}}-l_{\text{origin}}$ nulls, we compose the null candidates of size $l_{\text{align}}-l_{\text{origin}}$ and sort them by the null probabilities. We fix the null positions taken from the candidates one by one and execute the bit consensus algorithm. We repeat this process until the resulting consensus is error-free or no more candidate is found.

With the fixed nulls in common, the aligned reads are converted to bit sequences. We store the bit positions which are not unanimous, calculate the bit probability per bit position based on frequency, compose the bit candidates, and sort them by the bit probabilities. The bit sequence from each candidate goes through error detection. The final error-free consensus sequences which are not included in $S_{1}$ are denoted by $S_{2}$ and fed into the outer decoder one by one sequentially following $S_{1}$. The residual EDOL reads which did not contribute to generate $S_{2}$ are denoted by $R_{\text{REDOL}}$.

**Figure 4.**
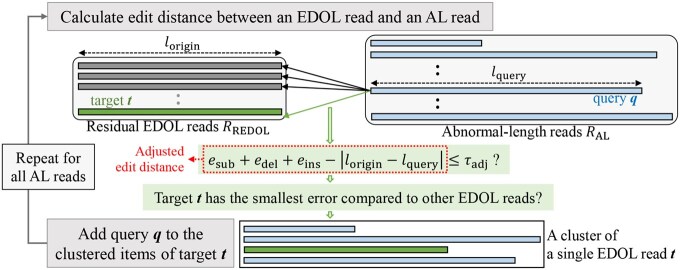
Tailored edit distance-based clustering algorithm for Stage 3. The edit distance between an EDOL read and an AL read is adjusted by subtracting the length difference $\left|l_{\text{origin}}-l_{\text{query}}\right|$. The parameter $l_{\text{query}}$ is the length of an AL read selected for the distance comparison. The threshold $\tau_{\text{adj}}$ can be tailored by error type for more refined clustering. A cluster consists of a single EDOL read and multiple AL reads.

**Stage 3:** To further generate error-free sequences, we additionally use the AL reads denoted by $R_{\text{AL}}$ as well as $R_{\text{REDOL}}$, which have not been used in Stages 1 and 2 but include much information on the original oligo sequences. We designed a tailored edit distance-based clustering algorithm to match the AL reads with various lengths to a single EDOL read as shown in Fig. 4. We modified the starcode algorithm to identify the numbers of substitutions, deletions, and insertions denoted by $e_{\text{sub}}$, $e_{\text{del}}$, and $e_{\text{ins}}$, respectively. Unlike substitution errors, indel errors do not incur erroneous consensus sequences if the reads are correctly aligned, so the portion of deletions or insertions in the total edit distance between reads of different lengths is excluded for the edit distance-based clustering. In the algorithm, reads are clustered when the adjusted edit distances between the reads do not exceed a given threshold $\tau_{\text{adj}}$. We also implemented an option to set a threshold for each type of error, allowing users to tailor the clustering process more precisely. The clusters generated by the proposed clustering also go through the multiple sequence alignment and the CAPMB consensus algorithm used in Stage 2. The extra error-free sequences generated in Stage 3 are denoted by $S_{3}$ and fed into the outer decoder in the same manner.

## 3 Results

We present the statistical profiles of sequence reads from our experiment. We also evaluate the effectiveness of the extra NPF reads and our sequence analysis method through decoding results.

### 3.1 Sequence read profile

From the sequencing experiment, we obtain paired-end 2 746 375 PF reads and 325 523 NPF reads from six tiles which are defined subsections of the flow cell surface used for Illumina sequencing. We calculated the ratio of successfully merged reads to the total reads as shown in Table 1. About 91.06% of PF reads and 64.02% of NPF reads were successfully merged. Among the total reads, 82.40% of PF reads and 56.37% of NPF reads have the original length after merging. We define the target length to be from 145 to 153 for our experiment. The error rates for target-length reads are shown in Table 1. NPF reads have a much higher substitution error rate but slightly higher insertion and deletion error rates than PF reads. Compared to the previous DNA research (Welzel *et al.* 2023), the error rates in this experiment are among the lowest reported (Supplementary Material S4, available as supplementary data at *Bioinformatics* online).

**Table 1.** Merged-read distributions by length and average base error rate.

Read type	Read distribution by length (nt)						Error rate of target-length reads			
	Failure	< 145	145–151	152	153	> 153	Sub	Del	Ins	Total
PF	8.94%	4.22%	2.80%	82.40%	1.01%	0.62%	0.09%	0.05%	0.01%	0.16%
NPF	35.98%	3.61%	1.89%	56.37%	0.65%	1.50%	3.84%	0.08%	0.04%	3.96%

**Figure 5.**
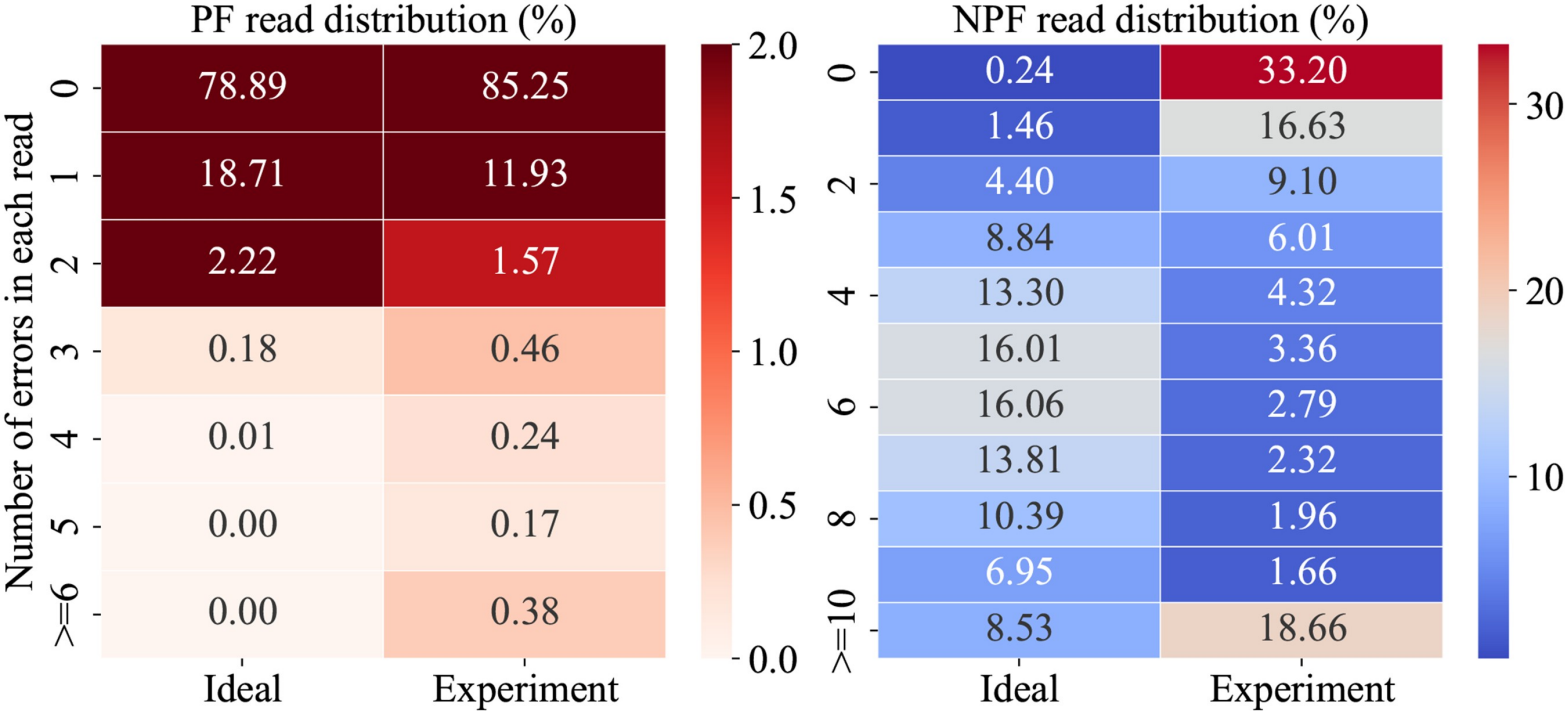
Comparison of the target-length read errors with the ideal distribution.

As mentioned before, indel errors are natural for the AL reads and not fatal during multiple sequence alignment and consensus so the substitution error rate is important to determine the reliability of the reads. The NPF reads have much higher base error rates than the PF reads and could be seen useless, but the decoding experiment will show that the extra use of NPF reads helps generate more error-free sequences. The reason is that NPF reads possess a characteristic where errors are concentrated in specific reads as shown in Fig. 5. Erroneous NPF reads are 4.53 times more frequent than erroneous PF reads. Figure 5 compares the distribution of the number of errors in the experiment with the binomial distribution. PF and NPF reads contain not only many error-free reads but also a significant number of high-error reads exceeding six and 10 errors, respectively.

Due to the risk of error propagation, high-error reads are more appropriately treated as dropouts detected rather than corrected. The combination of an inner detection code and an outer erasure code is a cost-effective and reliable approach for handling low base-level errors while correcting dropouts as erasures. This coding scheme is also well-suited for detecting NPF reads which especially have a high rate of substitutions. Despite the high-error rates of NPF reads in Table 1 and Fig. 5, 39.42% of NPF reads include five or less errors and can have potential for error correction through sequence analysis.

To further show error statistics of our experiment, we analyse the distribution of error runs in Table 2. The probability of an error run of a specific length is calculated as the ratio of the number of occurrences of that run length to the total number of error events. Unlike other error types which predominantly occur as single errors, deletions frequently appear in longer sequences. This suggests that deletion errors tend to occur in bursts within a read whereas other errors occur randomly. We also provide error characteristics including error rates per position and error correlations before and after merging in Supplementary Material S4, available as supplementary data at *Bioinformatics* online.

**Table 2.** Distribution of error runs in target-length reads.

Error type	Length of error runs in PF read				Length of error runs in NPF read			
	1	2	3–5	≥ 6	1	2	3–5	≥ 6
Sub	94.73%	3.71%	1.40%	0.17%	84.89%	11.91%	3.15%	0.06%
Del	74.67%	12.12%	9.97%	3.23%	82.58%	10.13%	5.83%	1.46%
Ins	98.62%	1.26%	0.11%	0.00%	97.37%	2.47%	0.17%	0.00%

### 3.2 Decoding with random sampling

To show whether low-quality reads are useful in practice for DNA data storage, we set up the following three scenarios: Erlich-PF, Erlich-ExtraNPF, and Prop-ExtraNPF. In Erlich-PF and Erlich-ExtraNPF, error-free sequences are obtained using Erlich’s sequence analysis method (Erlich and Zielinski 2017) which is the same with Stage 1 in our proposed workflow. Erlich’s method is described in Supplementary Material S2, available as supplementary data at *Bioinformatics* online. In Erlich-PF, only PF reads are used and in Erlich-ExtraNPF, both PF and NPF reads are used. Prop-ExtraNPF uses both PF and NPF reads to perform the proposed sequence analysis workflow shown in Fig. 2. We conducted decoding experiments to determine whether data are perfectly recovered with randomly sampled reads to compare the three scenarios. After sampling a fixed number of PF and NPF reads, we performed the preprocessing and the corresponding sequence analysis and checked whether LT erasure decoding succeeded or failed. In the Prop-ExtraNPF scenario, the edit distance thresholds $\tau_{e}$ and $\tau_{\text{adj}}$ were set to 5 and 4, respectively (Supplementary Material S3, available as supplementary data at *Bioinformatics* online). Based on the observation from Table 1, we used the AL reads of lengths from 145 to 153.

Performance gain is defined as the reduction ratio in the minimum number of randomly sampled reads required for perfect data recovery compared to the baseline scenario. Due to the high cost of DNA synthesis, most works minimize the number of sequenced reads required for perfect recovery instead of maximizing the data size synthesized. We conducted 36 independent data recovery experiments with random sampling. On average, Prop-ExtraNPF and Erlich-ExtraNPF reduced the reading costs for perfect data recovery by 6.83% and 3.96%, respectively, and achieved a maximum reduction of 19.67%.

**Figure 6.**
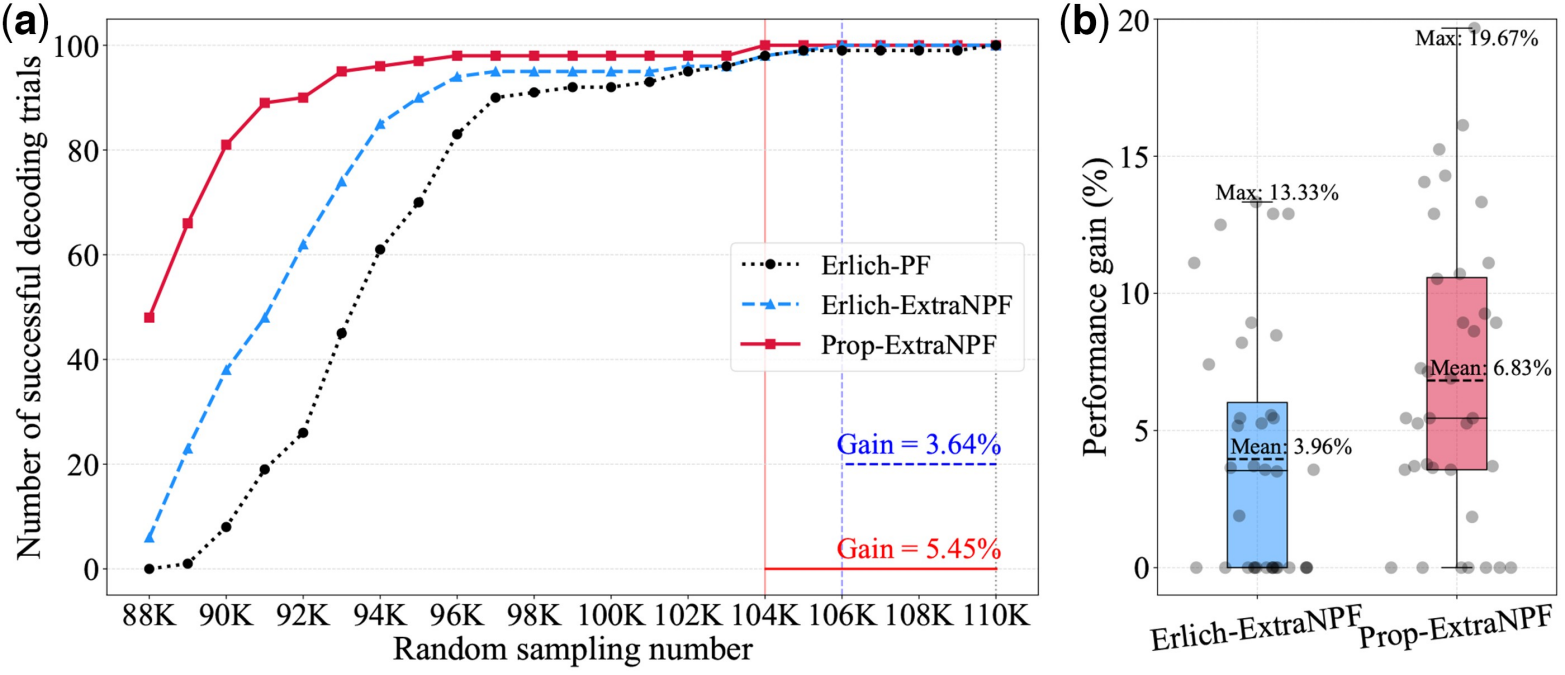
(a) Comparison of decoding results according to random sampling counts based on average data recovery performance gain. (b) Data recovery performance gain comparison under varying random seeds.

We presented one of the data recovery experiments near average in Fig. 6a. In this case, perfect decoding was achieved at random sampling numbers 104 000 for Prop-ExtraNPF, 106 000 for Erlich-ExtraNPF, and 110 000 for Erlich-PF. Prop-ExtraNPF and Erlich-ExtraNPF reduced the reading costs for perfect data recovery by 5.45% and 3.64%, respectively. Table 3 summarizes the analysis results for all scenarios at random sampling number 104 000, which achieves the perfect data recovery by Prop-ExtraNPF. In Stage 1, Erlich-ExtraNPF achieved a gain of 56.67 error-free sequences over Erlich-PF. In Stage 2, 34.62% of the input reads $R_{\text{EDOL}}$ were clustered, and 19.23% of the clusters have size 2 or larger and 98.84% of these clusters produced error-free sequences through the CAPMB consensus. Most of the consensus sequences duplicated with $S_{1}$ and 1.97% of them were distinct as error-free sequences $S_{2}$. In Stage 3, $R_{\text{REDOL}}$ from Stage 2 and $R_{\text{AL}}$ were clustered by the tailored edit distance-based clustering and the portion was 22.38%. Among them, 11.80% of the clusters have size of 2 or larger and 98.24% of these clusters generated error-free sequences through the CAPMB consensus. Approximately 2.18% of the error-free sequences were determined to be $S_{3}$ by removing the duplicated sequences. The error-free generation ratio of the CAPMB consensus in Stages 2 and 3 exceeds 98% and it indicates that the proposed consensus algorithm has high reliability. We also provide the comparison of sequence coverage and dropout with ideal Poission distribution in Supplementary Material S3, available as supplementary data at *Bioinformatics* online.

**Table 3.** Results of the Prop-ExtraNPF averaged over 100 trials at random sampling number.

		PF	PF + NPF
Stage 1 (Erlich’s method)	Number of merged reads	86324.68	93530.88
	Number of error-free sequences	17349.73	17404.22
	Number of error clusters	7577.01	11674.83
	Error-free sequence gain compared to using only PF reads		+56.67
Stage 2 (Proposed method)	Number of $R_{\text{EDOL}}$		11455.19
	Number of all clusters		9271.66
	Number of clusters of size 2 or larger		1782.47
	Number of corrected clusters by CAPMB consensus		1761.86
	Number of error-free sequences $S_{2}$		+34.78
Stage 3 (Proposed method)	Number of $R_{\text{REDOL}}$		7530.92
	Number of $R_{\text{AL}}$		4921.52
	Number of all clusters		10958.76
	Number of clusters of size 2 or larger		1293.53
	Number of corrected clusters by CAPMB consensus		1270.74
	Number of error-free sequences $S_{3}$		+27.64

The performance gain of each experiment is depicted in Fig. 6b and experimental settings are provided in Supplementary Table S5, available as supplementary data at *Bioinformatics* online. It is noted that in Fig. 6b, some random experiments seem not to show any performance gain of random sampling number for perfect recovery over Erlich-PF but a gain becomes evident when the number of decoding trials increases.

We compared the execution time using a single thread in a case of random sampling based on the achievement of perfect data recovery in each scenario. Erlich-ExtraNPF and Prop-ExtraNPF require 1.49% and 2.30% increase of the execution times over Erlich-PF, respectively (Supplementary Material S3, available as supplementary data at *Bioinformatics* online). Prop-ExtraNPF can be seen as a post-processing for Erlich-ExtraNPF and results in a short execution time. The use of extra NPF reads improves the recovery performance with a small increase of computational time.

## 4 Discussion and conclusion

In this article, we proposed a novel approach to improve the data recovery performance for DNA storage by leveraging low-quality reads previously overlooked. We observed that NPF reads which do not pass Illumina’s chastity filter have high-error rates but still contain valuable informations for generating error-free sequences. We also analysed that indel errors do not impair the generation of error-free sequences when combined with sequence analysis.

To enhance recovery performance, we elaborately designed a progressive sequence analysis workflow that maximizes the utilization of low-quality reads and generates additional error-free sequences. We introduced the CAPMB consensus algorithm which reliably produces error-free sequences from erroneous reads by utilizing error correction/detection codes and probabilistic majority, achieving high accuracy over 98%. We also developed a tailored edit distance-based clustering algorithm that adapts to variations in sequence length and the count of each error type to accommodate abnormal-length reads.

By integrating low-quality reads and the proposed workflow, we reduced reading costs by an average of 6.83% and up to 19.67% with variability, while maintaining writing costs. We demonstrated that NPF reads and abnormal-length reads can be systematically incorporated into DNA storage systems to extend the pool of usable data without compromising accuracy. The findings suggest the potential of low-quality reads to enhance DNA storage without the need for additional sequencing. Our workflow is broadly applicable to DNA data storage systems which use inner and outer codes and contributes to the development of high reliability, cost-effective, and scalable DNA data storage solutions.

## Supplementary Material

btaf335_Supplementary_Data

## Data Availability

The source codes and raw data are available at https://github.com/PParkJy/SAD-DNAstorage, and the current version used in this study is archived at https://doi.org/10.5281/zenodo.15571858.
