# Supplementary material for: Sequence analysis and decoding with extra low-quality reads for DNA data storage
Source: Bioinformatics. 2025 Jun 10;41(6):btaf335. doi: 10.1093/bioinformatics/btaf335 (PMC12187058; doi:10.1093/bioinformatics/btaf335)
Supplement: btaf335_Supplementary_Data [file btaf335_supplementary_data.pdf]

Supplementary Material

S1. Experimental information

Table S1. Sequence patterns used in DNA data storage experiments

Type	Sequence pattern
Adapter (Forward, 26nt)	GTTCAGAGTTCTACAGTCCGACGATC
Adapter (Reverse, 21nt)	GGAATTCTCGGGTGCCAAGG
Primer (RP1)	5' AATGATACGGCGACCACCGAGATCTACACGTTCAGAGTTCTACAGTCCGA 3'
Primer (RP1I)	5' CAGCAGAAGACGGCATACGAGATCGTGATGTGACTGGAGTTCCTTGGCACCCGAGAATTCCA TTGGCACCCGAGAATTCCA 3'
Index adapter	ATCACG

· Synthesis

18,000 oligo sequences are produced with a length of 199nt in the encoding process.

Oligo synthesis was requested from Twist Bioscience and a 300ng of oligo pool was synthesized.

· PCR

The lyophilized PCR sample was diluted to 25 ng/μl by nuclease-free water (Invitrogen).

PCR was performed with the below conditions and thermocycling process.

After amplification, the PCR sample was purified using 50 μl (1X) of AMPure XP (Beckman Coulter) and eluted with 20 μl of nuclease-free water (Invitrogen).

Table S2. PCR experiment conditions

Oligo pool sample (25ng/ul)	1μl (25ng)
RP1 primer(10uM)	2.5μl
RPI1 primer(10uM)	2.5μl
Q5 master mix*	25μl
Nuclease free water	19μl
Total	50μl

* Q5® Hot Start High-Fidelity 2X Master Mix

Table S3. Thermocycling cycles of PCR

98°C	98°C	60°C	72°C	72°C
30sec	10sec	30sec	30sec	5min

* The highlighted area is repeated for 10 cycles

· Sequencing

The PCR sample and Phi-X control v3 were denatured and diluted according to the Illumina guide to prepare a 10 pM library.

MiSeq Reagent Kit v3 (600-cycle) was used in sequencing with a 30% Phi-X control sample spike-in.

S2. Erlich's sequence analysis[R15]

As mentioned in the paper, we utilized Erlich's sequence analysis method as Stage 1 of our proposed method. Erlich and his team performed length filtering and sequence clustering. First, R1 (Forward) and R2 (Reverse) reads are selected and merged. If the length of the merged read is not equal to standard length *l*, it is discarded. Only reads with standard length are used in clustering. Erlich's clustering is the task of making only reads having the same bases into one cluster. After sorting the representative sequences in descending order of cluster size, the sequences are input one by one into the error detector and decoder in this order. Entering the order according to the cluster size is for the reliability of the sequence.

S3. Simulation results

S3.1. Dataset

We used 3,071,898 paired-end reads for our simulation. Among them, 2,746,375 were PF reads, and 325,523 were NPF reads. The read data are available as FASTQ files at https://github.com/PParkJy/SAD-DNAstorage.

S3.2. Edit distance threshold

In the paper, the edit distance-based clustering methods that we used and proposed allow the user to select the edit distance threshold. We calculated how many error-free sequences could be obtained using target-length reads with lengths ranging from 145 to 153. Sequence analysis was attempted 100 times with a random sampling number of 100,000, and the edit distance which yielded the highest average number of error-free sequences was selected. In both cases, the most error-free sequences were obtained when τ_e was set to 5 and τ_{adj} was set to 4 (shown in Table S4).

Table S4. Comparison of the number of error-free sequences based on the choice of edit distance

Edit distance		τ_{adj}							
		PF				PF + NPF			
		2	3	**4**	5	2	3	**4**	5
τ_e	2	17331.21	17331.75	17331.95	17332.07	17409.33	17410.92	17411.96	17412.68
	3	17333.34	17331.93	17334.07	17334.19	17413.52	17413.41	17416.14	17416.86
	4	17333.78	17334.32	17332.91	17334.63	17415.35	17416.93	17416.07	17418.68
	**5**	17334.02	17334.56	**17334.75**	17333.27	17416.84	17418.4	**17419.43**	17418.45

S3.3. Decoding results

The performance gain is expressed as a percentage, calculated by subtracting the ratio of the random sampling number that achieved perfect decoding in each scenario from the random sampling number that achieved perfect decoding in the Erlich-PF scenario from 1. The number of perfect decoding trials is 100.

We conducted multiple decoding experiments depending on seeds of the random number generator. In Table S5, we provide the decoding performance based on the random seeed of random number generator. On average, Prop-ExtraNPF and Erlich-ExtraNPF reduced the reading costs for perfect data recovery by 6.83% and 3.96s%, respectively, and achieved a maximum reduction of 19.67%.

Table S5. Decoding performance comparison based on random seed

Seed	Minimum Random sampling number when success perfect data recovery			Performance Gain	
	Erlich-PF	Erilch-ExtraNPF	Prop-ExtraNPF	Erilch-ExtraNPF	Prop-ExtraNPF
97119	114000	108000	108000	5.26%	5.26%
97219	102000	102000	102000	0.00%	0.00%
97319	112000	112000	100000	0.00%	10.71%
97419	118000	108000	100000	8.47%	15.25%
97519	124000	108000	104000	12.90%	16.13%
97619	116000	110000	106000	5.17%	8.62%
97719	108000	102000	98000	5.56%	9.26%
97819	116000	116000	108000	0.00%	6.90%
98119	128000	112000	110000	12.50%	14.06%
98219	110000	110000	110000	0.00%	0.00%
98419	110000	106000	104000	3.64%	5.45%
99619	110000	104000	104000	5.45%	5.45%
99719	112000	102000	102000	8.93%	8.93%
99819	120000	104000	104000	13.33%	13.33%
99919	114000	114000	108000	0.00%	5.26%
100019	110000	110000	110000	0.00%	0.00%
100119	114000	110000	102000	3.51%	10.53%
100419	108000	100000	96000	7.41%	11.11%
100519	112000	108000	102000	3.57%	8.93%
100619	106000	104000	102000	1.89%	3.77%
100719	112000	112000	104000	0.00%	7.14%
100819	108000	104000	104000	3.70%	3.70%
101919	112000	112000	112000	0.00%	0.00%
102019	110000	104000	104000	5.45%	5.45%
102319	112000	108000	108000	3.57%	3.57%
102519	122000	112000	98000	8.20%	19.67%
102619	108000	108000	106000	0.00%	1.85%
102719	104000	104000	104000	0.00%	0.00%

102819	110000	110000	106000	0.00%	3.64%
103119	108000	108000	104000	0.00%	3.70%
103219	112000	112000	112000	0.00%	0.00%
103319	110000	110000	110000	0.00%	0.00%
103419	110000	110000	102000	0.00%	7.27%
103519	112000	112000	108000	0.00%	3.57%
104319	126000	112000	108000	11.11%	14.29%
105019	124000	108000	108000	12.90%	12.90%
**Average**	**112888.89**	**108222.22**	**104944.44**	**3.96%**	**6.83%**

The number of successful decodings based on performance gain for all scenarios is shown in Tables S6-S8.

Table S6. Comparison of the number of successful decodings when the performance gain is average

**Seed: 98419 (Near-Average case)**			
**Random Sampling Number**	**Erlich-PF**	**Erlich-ExtraNPF**	**Prop-ExtraNPF**
87000	0	3	32
88000	0	6	48
89000	1	23	66
90000	8	38	81
91000	19	48	89
92000	26	62	90
93000	45	74	95
94000	61	85	96
95000	70	90	97
96000	83	94	98
97000	90	95	98
98000	91	95	98
99000	92	95	98
100000	92	95	98
101000	93	95	98
102000	95	96	98
103000	96	96	98
**104000**	98	98	**100**
105000	99	99	100
**106000**	99	**100**	100
107000	99	100	100
108000	99	100	100
109000	99	100	100
**110000**	**100**	100	100

Table S7. Comparison of the number of successful decodings when the performance gain is high

**Seed: 100419**			
**Random sampling number**	**Erlich-PF**	**Erlich-ExtraNPF**	**Prop-ExtraNPF**
87000	0	6	46

88000	1	15	54
89000	2	29	66
90000	9	46	76
91000	20	58	82
92000	25	67	85
93000	38	70	91
94000	49	79	93
95000	62	86	99
**96000**	72	93	**100**
97000	82	96	100
98000	88	97	100
99000	92	99	100
**100000**	92	**100**	100
101000	96	100	100
102000	98	100	100
103000	98	100	100
104000	98	100	100
105000	99	100	100
106000	99	100	100
**107000**	**100**	100	100

Table S8. Comparison of the number of successful decodings when the performance gain of Erlich-ExtraNPF is 0

**Seed: 103419**			
**Random Sampling Number**	**Erlich-PF**	**Erlich-ExtraNPF**	**Prop-ExtraNPF**
87000	0	7	40
88000	1	10	53
89000	4	24	69
90000	8	39	79
91000	20	56	84
92000	27	67	87
93000	40	76	91
94000	58	82	93
95000	69	88	96
96000	72	92	97
97000	82	94	97
98000	91	96	98
99000	95	97	98
100000	97	97	98
101000	98	98	99
**102000**	99	99	**100**
103000	99	99	100
104000	99	99	100
105000	99	99	100
106000	99	99	100
107000	99	99	100
108000	99	99	100

**109000**	**100**	**100**	100

S3.4. Sequence analysis results

Table 3 in the paper present the sequence analysis results for the Near-average case. These results are represented in detail in Tables S9 and S10 below. The results with other random seeds are summarized in Tables S11-S14. The random sampling numbers used as the basis for the result analysis represent the number of times the proposed method achieved perfect decoding.

Table S9. Result of Erlich's sequence analysis at random sampling number 106,000 (Near-Average case)

RSN = 106,000		PF	PF + NPF
**Stage 1**	Number of merged reads	86324.68	93530.88
	Number of error-free sequences (S_1)	17349.73	17404.22
	Number of error clusters	7557.01	11674.83
	Ratio of error-free sequences (S_1) to all clusters	69.66%	59.85%
	Ratio of error clusters to all clusters	30.34%	40.15%
	Error-free sequence gain compared to using only PF reads		54.49
	Ratio of error-free sequence gain compared to using only PF reads		0.31%

Table S10. Result of proposed sequence analysis at random sampling number 104,000 (Near-Average case)

RSN = 104,000		PF	PF + NPF
**Stage 1**	Number of merged reads	86324.68	93530.88
	Number of error-free sequences (S_1)	17349.73	17404.22
	Number of error clusters	7557.01	11674.83
	Ratio of error-free sequences (S_1) to all clusters	69.66%	59.85%
	Ratio of error clusters to all clusters	30.34%	40.15%
	Error-free sequence gain compared to using only PF reads		56.67
	Ratio of error-free sequence gain compared to using only PF reads		0.33%
**Stage 2**	Number of EDOL reads before clustering		11455.19
	Number of all clusters		9271.66
	Number of clusters of size 2 or larger		1782.47
	Number of corrected clusters by CAPMB consensus		1761.86
	Number of uncorrected clusters by CAPMB consensus		20.61
	Number of reads in uncorrected clusters		41.37
	Number of duplicated clusters with S_1		1727.08
	Number of error-free clusters (S_2) (duplications were removed)		34.78
	Ratio of clusters of size 2 or larger to all clusters		19.23%
	Ratio of corrected clusters to size 2 or larger clusters		98.84%
	Ratio of error-free clusters in corrected clusters		1.97%
	Ratio of uncorrected clusters to size 2 or larger clusters		1.16%
	Ratio of error-free sequence gain compared to using only PF reads		0.20%
**Stage 3**	Number of REDOL reads (R_{REDOL}) before clustering		7530.92
	Number of AL reads (R_{AL}) before clustering		4921.52
	Number of all clusters		10958.76
	Number of clusters of size 2 or larger		1293.53
	Number of corrected clusters by CAPMB consensus		1270.74
	Number of uncorrected clusters by CAPMB consensus		22.79
	Number of reads in uncorrected clusters		45.78
	Number of duplicated clusters with S_1 and S_2		1243.1

	Number of error-free clusters (S_3) (duplications were removed)		27.64
	Ratio of clusters of size 2 or larger to all clusters		11.80%
	Ratio of corrected clusters to size 2 or larger clusters		98.24%
	Ratio of error-free clusters in corrected clusters		2.18%
	Ratio of uncorrected clusters to size 2 or larger clusters		1.76%
	Ratio of error-free sequence gain compared to using only PF reads		0.16%

Table S11. Result of Erlich's sequence analysis at random sampling number 100,000 (Seed: 100419)

RSN = 100,000		PF	PF + NPF
Stage 1	Number of merged reads	81424.74	88218.10
	Number of error-free sequences (S_1)	17238.25	17299.99
	Number of error clusters	7140.83	11023.53
	Ratio of error-free sequences (S_1) to all clusters	70.71%	61.08%
	Ratio of error clusters to all clusters	29.29%	38.92%
	Error-free sequence gain compared to using only PF reads		67.24
	Ratio of error-free sequence gain compared to using only PF reads		0.36%

Table S12. Result of proposed sequence analysis at random sampling number 96,000 (Seed: 100419)

RSN = 96,000		PF	PF + NPF
Stage 1	Number of merged reads	78169.33	84690.04
	Number of error-free sequences (S_1)	17150.5	17217.74
	Number of error clusters	6854.41	10580.04
	Ratio of error-free sequences (S_1) to all clusters	71.45%	61.94%
	Ratio of error clusters to all clusters	28.55%	38.06%
	Error-free sequence gain compared to using only PF reads		41.16
	Ratio of error-free sequence gain compared to using only PF reads		0.39%
Stage 2	Number of EDOL reads before clustering		10580.04
	Number of all clusters		8694.24
	Number of clusters of size 2 or larger		1563.62
	Number of corrected clusters by CAPMB consensus		1544.97
	Number of uncorrected clusters by CAPMB consensus		18.65
	Number of reads in uncorrected clusters		37.47
	Number of duplicated clusters with S_1		1503.81
	Number of error-free clusters (S_2) (duplications were removed)		41.16
	Ratio of clusters of size 2 or larger to all clusters		17.99%
	Ratio of corrected clusters to size 2 or larger clusters		98.81%
	Ratio of error-free clusters in corrected clusters		2.66%
	Ratio of uncorrected clusters to size 2 or larger clusters		1.19%
	Ratio of error-free sequence gain compared to using only PF reads		0.24%
Stage 3	Number of REDOL reads (R_{REDOL}) before clustering		7168.53
	Number of AL reads (R_{AL}) before clustering		4537.6
	Number of all clusters		10375.59
	Number of clusters of size 2 or larger		1162.8
	Number of corrected clusters by CAPMB consensus		1143.51
	Number of uncorrected clusters by CAPMB consensus		19.29
	Number of reads in uncorrected clusters		38.69
	Number of duplicated clusters with S_1 and S_2		1111.18

	Number of error-free clusters (S_3) (duplications were removed)		32.33
	Ratio of clusters of size 2 or larger to all clusters		11.21%
	Ratio of corrected clusters to size 2 or larger clusters		98.34%
	Ratio of error-free clusters in corrected clusters		2.83%
	Ratio of uncorrected clusters to size 2 or larger clusters		1.66%
	Ratio of error-free sequence gain compared to using only PF reads		0.19%

Table S13. Result of Erlich's sequence analysis at random sampling number 109,000 (Seed: 103419)

RSN = 109,000		PF	PF + NPF
Stage 1	Number of merged reads	88749.71	96154.48
	Number of error-free sequences (S1)	17399.71	17449.89
	Number of error clusters	7765.19	11989.31
	Ratio of error-free sequences (S1) to all clusters	69.14%	59.27%
	Ratio of error clusters to all clusters	30.86%	40.73%
	Error-free sequence gain compared to using only PF reads		50.18
	Ratio of error-free sequence gain compared to using only PF reads		0.29%

Table S14. Result of proposed sequence analysis at random sampling number 102,000 (Seed: 103419)

RSN = 102,000		PF	PF + NPF
Stage 1	Number of merged reads	83052.14	89981.94
	Number of error-free sequences (S_1)	17278.28	17336.41
	Number of error clusters	7266.24	11221.46
	Ratio of error-free sequences (S_1) to all clusters	70.40%	60.71%
	Ratio of error clusters to all clusters	29.60%	39.29%
	Error-free sequence gain compared to using only PF reads		58.13
	Ratio of error-free sequence gain compared to using only PF reads		0.34%
Stage 2	Number of EDOL reads before clustering		11221.46
	Number of all clusters		9119.07
	Number of clusters of size 2 or larger		1722.96
	Number of corrected clusters by CAPMB consensus		1704.04
	Number of uncorrected clusters by CAPMB consensus		18.92
	Number of reads in uncorrected clusters		37.99
	Number of duplicated clusters with S_1		1666.65
	Number of error-free clusters (S_2) (duplications were removed)		37.39
	Ratio of clusters of size 2 or larger to all clusters		18.89%
	Ratio of corrected clusters to size 2 or larger clusters		98.90%
	Ratio of error-free clusters in corrected clusters		2.19%
	Ratio of uncorrected clusters to size 2 or larger clusters		1.10%
	Ratio of error-free sequence gain compared to using only PF reads		0.22%
Stage 3	Number of REDOL reads (R_{REDOL}) before clustering		7434.34
	Number of AL reads (R_{AL}) before clustering		4813.24
	Number of all clusters		10800.55
	Number of clusters of size 2 or larger		1257.92
	Number of corrected clusters by CAPMB consensus		1234.52
	Number of uncorrected clusters by CAPMB consensus		23.4
	Number of reads in uncorrected clusters		46.95
	Number of duplicated clusters with S_1 and S_2		1206.02
	Number of error-free clusters (S_3) (duplications were removed)		28.5

	Ratio of clusters of size 2 or larger to all clusters		11.65%
	Ratio of corrected clusters to size 2 or larger clusters		98.14%
	Ratio of error-free clusters in corrected clusters		2.31%
	Ratio of uncorrected clusters to size 2 or larger clusters		1.86%
	Ratio of error-free sequence gain compared to using only PF reads		0.16%

S3.5. Coverage and Dropout

To assess the quality of the sequencing data when perfect data recovery is ach, we calculated the distribution of coverage as shown in Figure S1. First, we performed a random sampling of 110,000 reads and conducted the preprocessing described in Section 2.3.1 of the paper. The random sampling number is the number that achieved perfect decoding in the best case of Erlich-PF scenario (Section S3.4.). Afterward, we matched the merged reads to the oligo sequences following the same method outlined in the section S4.1. Coverage is calculated by determining how many times a given oligo sequence was sequenced. In the case of 110,000 reads and 18,000 oligo sequences, the ideal coverage distribution forms an ideal Poisson distribution with a mean of approximately 6.11. As shown in Figure S1, both PF and ExtraNPF exhibit a leftward shift, meaning lower coverage compared to the ideal distribution. The primary reason is that some reads fail to merge during the paired-end read merging process in preprocessing. (Approximately 9% of PF reads and 36% of NPF reads fail to merge.) Nevertheless, ExtraNPF which uses both PF and NPF reads was closer to the ideal coverage distribution compared to PF alone.

Additionally, we compared the dropout rate relative to 18,000 oligos. In an ideal scenario, only 0.22% of the oligos should have been lost. However, the observed dropout rates were 1.18% for ExtraNPF and 1.61% for PF, corresponding to 5.31 times and 7.24 times the ideal dropout rate, respectively. Nevertheless, ExtraNPF reduced the dropout rate by approximately 1.36 times compared to using only PF read.

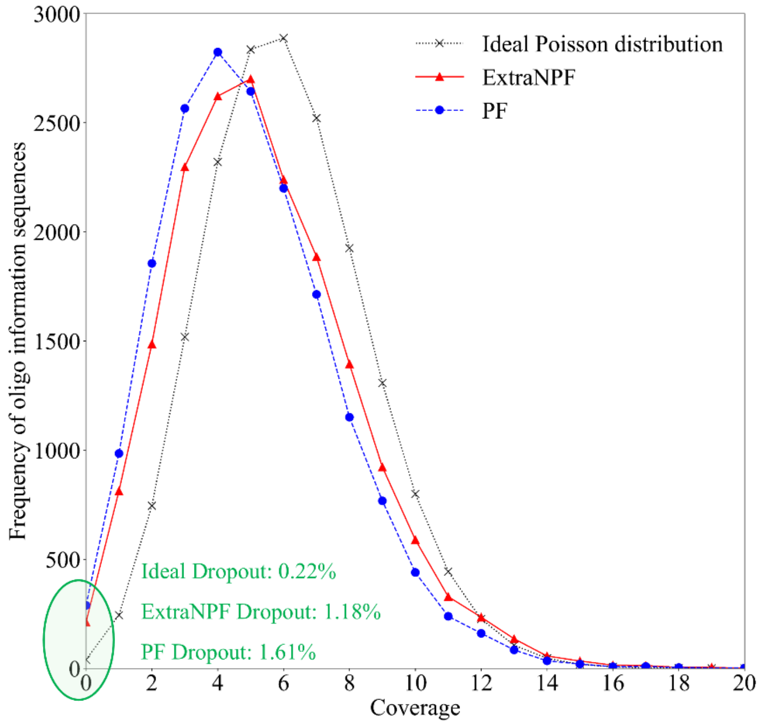

Figure S1. Comparison of the sequence coverage distribution and dropouts for 18,000 oligo sequences from 110,000 randomly sampled reads to the ideal Poisson distribution

S3.6. Execution time

We compared the execution time of three scenarios of Near-Average case when all 100 decoding trials were successfully completed. All scenarios were executed on an Ubuntu 18.04 OS with an Intel Xeon Silver 4210R CPU and 64GB RAM using a single thread. The execution times for each process are provided in Table S15. Erlich-ExtraNPF and Prop-ExtraNPF take 1.01 times and 1.02 times longer than Erlich-PF, respectively.

Table S15. Comparison of execution time of each scenario

Scenario	Process	Second	
Erlich-PF (RSN=110,000)	Random sampling	209	
	Merging	3195	
	Filtering PF and Original-length reads	81	
	Clustering and Error detection	871	
	Decoding	2277	
	Total	**6633**	
Erlich-ExtraNPF (RSN=106,000)	Random sampling	213	
	Merging	3070	
	Filtering Original-length reads	21	
	Clustering and Error detection	968	
	Decoding	2460	
	Total	**6732**	
Prop-ExtraNPF (RSN=104,000)	Random sampling	213	Stage 1
	Merging	3046	
	Original-Length filtering	18	
	Clustering and Error detection	969	
	Decoding	2398	
	Edit distance-based clustering	3	Stage 2
	In-cluster alignment	21	
	CAPMB	6	
	Filtering AL reads	1	Stage 3
	Tailored edit distance-based clustering	38	
	In-cluster alignment	16	
	CAPMB	3	
	Decoding	54	
	Total	**6786**	

S4. DNA data storage characteristics

In this section, we provide various error statistics that may assist in understanding the characteristics of the DNA storage channel.

S4.1. Method for error estimation

We estimated errors by performing one-to-one mapping between sequencing data and the original oligo sequences. The sequencing data used for error estimation included the paired-end reads—R1 (forward) and R2 (reverse)—as well as the merged reads obtained by overlapping R1 and R2 using PEAR of version 0.9.11 [R16]. Although the original length of R1 and R2 reads is 151 nt, previous sequencing studies have reported instability in the last base [R21-22]. Based on these findings, we excluded the final base. Therefore, the length of our R1 and R2 read is 150 nt.

Adapter trimming was performed on R1 and R2 reads using Fastp of version 0.20.1 [R17]. We modified the default Fastp algorithm to initiate trimming when a sequence similar to the adapter pattern (described in Section S1) was detected with at least 7 matching bases. As a result, the adapter-trimmed R1 and R2 reads have lengths of 143 nt or less, and no reads with lengths between 144 nt and 149 nt exist.

Mapping between reads and oligo sequences was performed based on edit distance by using the editops function from the python-Levenshtein package of version 0.20.9 (https://pypi.org/project/python-Levenshtein/). The mapping was conducted in the following order: First, the edit distances between each read (R1, R2, and merged) and all oligo sequences were calculated. Since R1 and R2 reads have a maximum length of 150 nt and are aligned to 152 nt oligo sequences, this differences introduce apparent deletions. To correct for this, we analyzed the error positions reported during edit distance computation and identified deletions occurring within the final two positions. We classified the deletions into length-related deletions and true deletions. The length-related deletions were excluded from the deletion count. Also, reads from which all bases were trimmed were considered to have 152 base deletions.

Second, if multiple oligo sequences yielded the same minimum edit distance for a given read, one was randomly selected. In cases where the same read (i.e., sharing the same cluster ID from the sequencing process) did not map to the same oligo sequence across R1, R2, and merged reads, the oligo sequence with the smallest edit distance among them was chosen. Subsequently, the edit distances for all reads were recalculated based on this selected oligo sequence.

S4.2. Error rate analysis according to read length

We analyzed substitution, deletion, and insertion error rates as a function of read length for R1, R2, and merged reads. Error rates are calculated by dividing the number of each error type by the product of the number of reads of the given length and the oligo sequence length (152nt).

As expected, the error rate is lowest when the read length is the original length. When the read length deviates from the original, the deletion or insertion error rate increases due to the length difference. Despite this, the substitution error rate is consistently lower than the other error rates. As discussed in the manuscript, this implies the potential usability of abnormal-length reads. Also, NPF reads show higher error rates than the PF reads and could be seen useless but the decoding experiment will show that the extra use of NPF reads helps generate more error-free sequences.

As well-established in previous sequencing studies, the error rate of R2 reads is generally higher than that of R1 reads [R18]. However, when R1 and R2 reads are merged, a significant reduction in the overall error rate can be observed. This demonstrates the error-correcting potential of paired-end sequencing.

Table S16. Length distribution and average base error rate of merged reads

Length (nt)	PF				NPF			
	Ratio	Error rate			Ratio	Error rate		
		Sub	Del	Ins		Sub	Del	Ins
**50–139**	2.87%	0.0069	0.3863	0.0009	2.65%	0.0281	0.3798	0.0023
**140–144**	0.27%	0.0111	0.0680	0.0025	0.21%	0.0539	0.0710	0.0081
**145**	0.07%	0.0116	0.0488	0.0029	0.05%	0.0443	0.0513	0.0074
**146**	0.08%	0.0099	0.0418	0.0024	0.05%	0.0461	0.0454	0.0075
**147**	0.10%	0.0090	0.0350	0.0022	0.06%	0.0463	0.0386	0.0065
**148**	0.13%	0.0066	0.0277	0.0014	0.09%	0.0339	0.0296	0.0038
**149**	0.21%	0.0040	0.0206	0.0009	0.15%	0.0390	0.0235	0.0041
**150**	0.49%	0.0023	0.0136	0.0004	0.33%	0.0269	0.0143	0.0013
**151**	2.80%	0.0011	0.0067	0.0001	1.89%	0.0238	0.0070	0.0004
**152***	82.40%	0.0009	0.00001	0.00001	56.37%	0.0390	0.0003	0.0003
**153**	1.01%	0.0018	0.0003	0.0069	0.65%	0.0265	0.0008	0.0075
**154–290**	0.62%	0.1444	0.0249	0.2075	1.50%	0.1722	0.0311	0.2075
**145–153****	87.30%	0.0009	0.0005	0.0001	59.66%	0.0384	0.0008	0.0004
**Total**	91.06%	0.0027	0.0131	0.0038	64.02%	0.0429	0.0182	0.0135

* Original length

** Target length used in Prop-ExtraNPF

Table S17. Length distribution and average base error rate of R1 reads

Length (nt)	PF				NPF			
	Ratio	Error rate			Ratio	Error rate		
		Sub	Del	Ins		Sub	Del	Ins
**< 140**	4.63%	0.00090	0.56389	0.00003	4.19%	0.00671	0.62883	0.00005

**140–143**	0.24%	0.00333	0.06869	0.00048	0.16%	0.05191	0.07144	0.00290
**150***	95.13%	0.02641	0.00860	0.00681	95.65%	0.15145	0.02919	0.02252
**Total**	-	0.02518	0.03447	0.00648	-	0.14524	0.05436	0.02155

* Original length of R1 reads

** Due to adapter trimming, no R1 reads with lengths between 144 nt and 149 nt are present.

*** Total ratio is 100%.

Table S18. Length distribution and average base error rate of R2 reads

**Length (nt)**	**PF**				**NPF**			
	**Ratio**	**Error rate**			**Ratio**	**Error rate**		
		**Sub**	**Del**	**Ins**		**Sub**	**Del**	**Ins**
**< 140**	4.39%	0.00080	0.55137	0.00002	3.46%	0.00818	0.61948	0.00003
**140–143**	0.22%	0.00927	0.07017	0.00154	0.13%	0.07688	0.07550	0.00631
**150***	95.39%	0.03026	0.00914	0.00713	96.41%	0.21042	0.02893	0.02167
**Total**	-	0.02893	0.03307	0.00681	-	0.20333	0.04941	0.02090

* Original length of R2 reads

** Due to adapter trimming, no R2 reads with lengths between 144 nt and 149 nt are present.

*** Total ratio is 100%.

S4.3. Frequency analysis of consecutive errors

To evaluate whether the error pattern is random or bursty, we analyzed the distribution of consecutive error run lengths within each read following the approach presented in Fig. 2d of [R19]. The proportion of each error run length to the total number of error run occurrences was presented for all merged reads as well as for R1 and R2 reads in the tables below. The table for target-length merged reads is included in the manuscript. Unlike other error types where single-base error runs dominate, deletions tend to occur as long patterns. This suggests that while substitutions and insertions occur randomly, deletions are more likely to occur in bursts.

Table S19. Distribution of error runs in all merged reads

**Error type**	**Length of error runs in PF read**				**Length of error runs in NPF read**			
	**1**	**2–3**	**4–6**	**> 6**	**1**	**2–3**	**4–6**	**> 6**
**Sub**	82.42%	16.39%	1.13%	0.05%	83.40%	15.68%	0.90%	0.02%
**Del**	40.60%	27.28%	14.44%	17.67%	45.89%	28.12%	14.04%	11.95%
**Ins**	88.28%	11.53%	0.19%	0.00%	88.55%	11.27%	0.18%	0.00%

Table S20. Distribution of error runs in R1 reads

**Error type**	**Length of error runs in PF read**				**Length of error runs in NPF read**			
	**1**	**2–3**	**4–6**	**> 6**	**1**	**2–3**	**4–6**	**> 6**
**Sub**	68.10%	28.52%	3.20%	0.18%	69.48%	26.75%	3.47%	0.30%
**Del**	57.49%	25.72%	8.28%	8.51%	66.87%	25.51%	4.22%	3.41%
**Ins**	92.87%	7.09%	0.04%	0.00%	93.22%	6.75%	0.04%	0.00%

Table S21. Distribution of error runs in R2 reads

**Error type**	**Length of error runs in PF read**				**Length of error runs in NPF read**			
	**1**	**2–3**	**4–6**	**> 6**	**1**	**2–3**	**4–6**	**> 6**
**Sub**	68.18%	28.14%	3.50%	0.18%	64.18%	26.74%	6.40%	2.68%
**Del**	57.29%	26.73%	8.01%	7.97%	65.31%	27.46%	4.15%	3.08%
**Ins**	92.84%	7.12%	0.04%	0.00%	93.00%	6.96%	0.04%	0.00%

S4.4. Distribution of errors per read

To determine how frequently substitution, deletion, and insertion errors occur within individual reads, we analyzed the distribution of errors per read using the same approach as in Fig. 2e of [R19]. In most cases, no errors occur. In cases where errors occur, they are predominantly single errors. As mentioned in Section S4.2., NPF reads with high error rates tend to have multiple substitution errors within a single read. As described in Section 4.3., deletion errors tend to occur in long patterns. This is supported by the observation of multiple deletions within individual reads, as shown in the table below.

Table S22. Error distribution of target-length merged reads

**Error type**	**The number of errors in PF reads**				**The number of errors in NPF reads**			
	**0**	**1**	**2–6**	**> 6**	**0**	**1**	**2–6**	**> 6**
**Sub**	**90.31%**	8.49%	1.04%	0.16%	**35.46%**	15.86%	24.39%	24.29%
**Del**	**95.50%**	3.24%	1.15%	0.10%	**93.11%**	4.71%	2.00%	0.19%
**Ins**	**98.76%**	1.21%	0.00%	0.03%	**96.35%**	2.66%	0.88%	0.11%

Table S23. Error distribution of all merged reads

**Error type**	**The number of errors in PF reads**				**The number of errors in NPF reads**			
	**0**	**1**	**2–6**	**> 6**	**0**	**1**	**2–6**	**> 6**
**Sub**	**89.76%**	8.27%	1.04%	0.93%	**35.38%**	15.37%	23.49%	25.77%
**Del**	**91.78%**	3.24%	1.32%	3.66%	**87.47%**	4.82%	2.66%	5.05%
**Ins**	**98.03%**	1.19%	0.07%	0.72%	**93.82%**	2.60%	1.03%	2.56%

Table S24. Error distribution of R1 reads

**Error type**	**The number of errors in PF reads**				**The number of errors in NPF reads**			
	**0**	**1**	**2–6**	**> 6**	**0**	**1**	**2–6**	**> 6**
**Sub**	**81.18%**	8.53%	2.17%	8.12%	12.99%	8.75%	21.28%	**56.97%**
**Del**	**79.88%**	2.87%	4.68%	12.57%	**42.54%**	3.54%	22.77%	31.16%
**Ins**	**90.74%**	1.23%	0.50%	7.53%	**68.21%**	2.77%	3.41%	25.60%

Table S25. Error distribution of R2 reads

**Error type**	**The number of errors in PF reads**				**The number of errors in NPF reads**			
	**0**	**1**	**2–6**	**> 6**	**0**	**1**	**2–6**	**> 6**
**Sub**	**75.66%**	10.08%	4.94%	9.32%	7.56%	5.64%	18.71%	**68.09%**
**Del**	**78.49%**	2.94%	6.05%	12.53%	**38.53%**	3.65%	29.59%	28.23%
**Ins**	**90.42%**	1.28%	0.57%	7.73%	**69.36%**	3.40%	3.40%	23.84%

S4.5. Comparison of edit distance distribution with the ideal binomial distribution

It is generally known that sequencing reads in DNA storage systems follow a binomial distribution [R20]. We constructed an ideal binomial distribution using the edit error rate (i.e., the sum of substitution, deletion, and insertion error rates) and the number of reads from Table S16 in Section S4.2. We then compared the edit distance distributions of target-length merged reads and all merged reads with this ideal distribution. The distribution for the target-length merged reads is included in the manuscript.

For both PF and NPF reads, we observed a higher number of error-free reads as well as high-error reads compared to the ideal binomial distribution. This indicates that errors in DNA storage systems do not occur uniformly across all reads as assumed in a binomial distribution, but are instead concentrated in a subset of reads. These high-error reads are relatively few and require substantial redundancy and complexity for error correction. Therefore, discarding them through detection is a more reasonable approach.

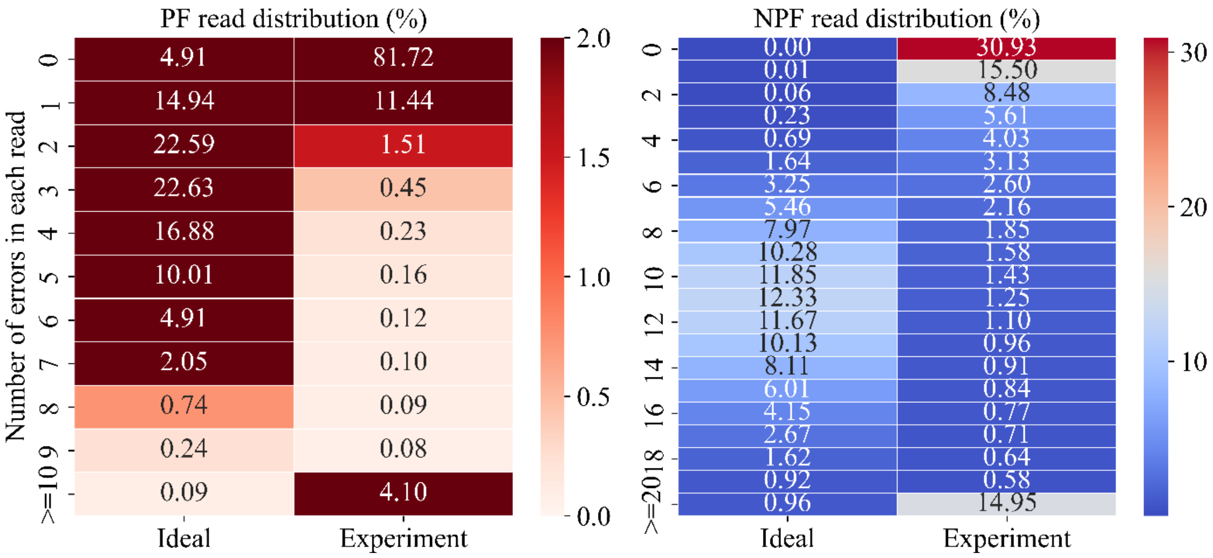

Figure S2. Comparison of the all merged read errors with the ideal distribution (Left: PF read, Right: NPF read)

S4.6. Analysis of correlations between error types

To investigate whether errors in DNA storage systems occur independently or in a correlated manner, we analyzed the proportion of reads according to different combinations of error types. The error combinations were classified into eight categories: No error (no errors present), Sub (only substitution errors), Del (only deletion errors), Ins (only insertion errors), Sub&Del (substitution and deletion), Sub&Ins (substitution and insertion), Del&Ins (deletion and insertion), and Sub&Del&Ins (all three types of errors).

For R1 and R2 reads, Ins cases do not occur because no reads are longer than the original oligo sequence. In R1 and R2 reads, the proportion of independently occurring errors (Sub, Del, Ins) and correlated errors does not differ significantly. And substitution errors tend to dominate the correlated-error cases and this is especially pronounced in NPF reads where substitution errors are frequently observed both independently and alongside other error types.

However, errors that occur together are often eliminated during the merging process of R1 and R2 reads. In merged reads, independent errors become more prominent than correlated errors. Reads with only deletion or insertion errors (Ins, Del reads) can potentially be useful if proper sequence clustering and alignment are performed because they have no substitution errors. These Ins and Del reads are particularly common in PF reads.

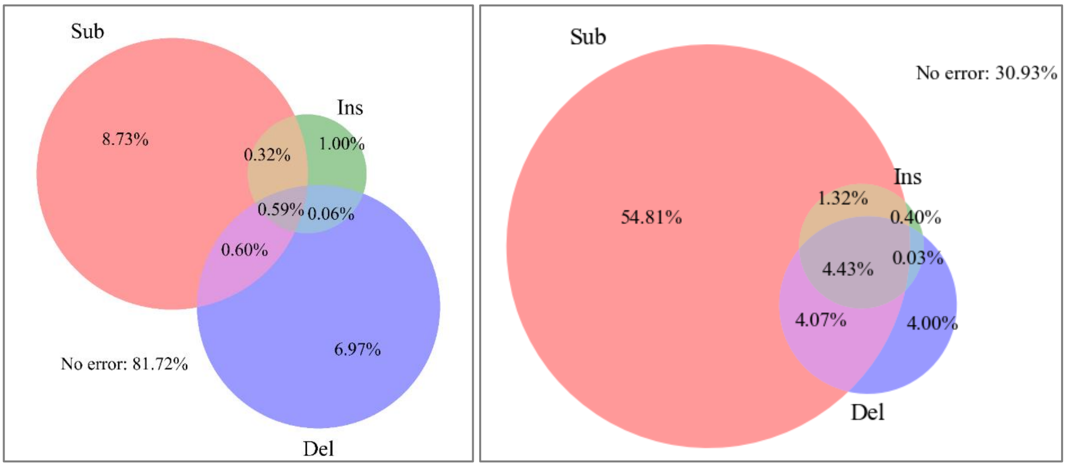

Figure S3. Read distribution by error combination of all merged reads (Left: PF read, Right: NPF read)

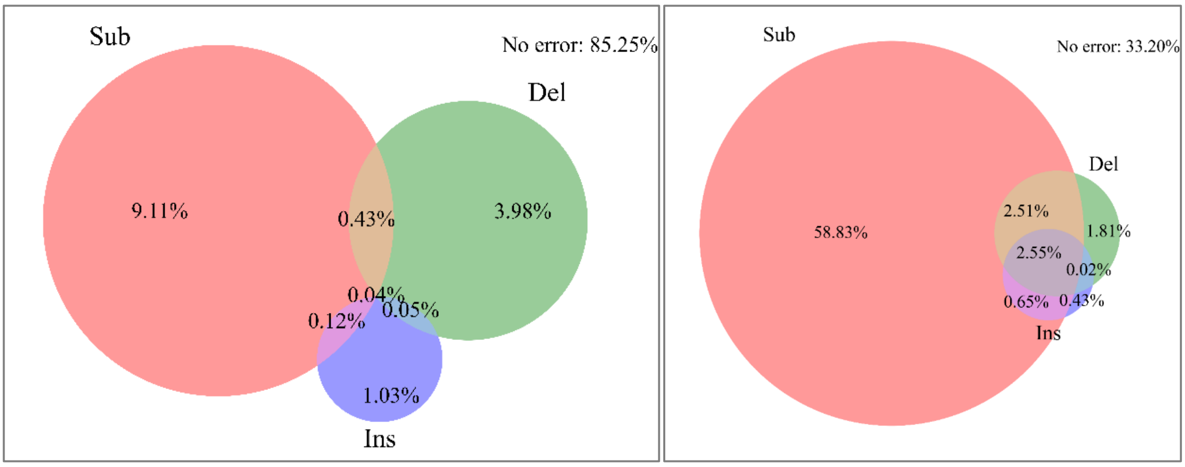

Figure S4. Read distribution by error combination of target-length merged reads (Left: PF read, Right: NPF read)

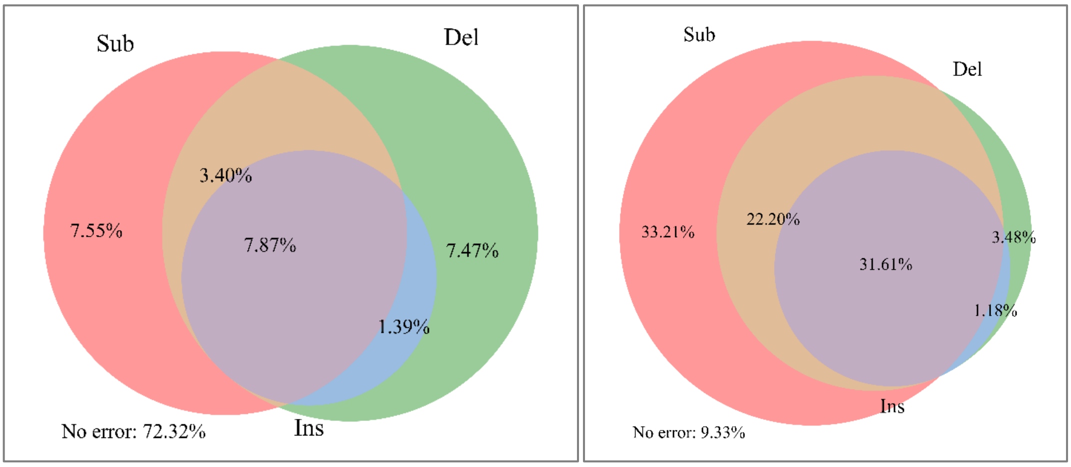

Figure S5. Read distribution by error combination of R1 reads (Left: PF read, Right: NPF read)

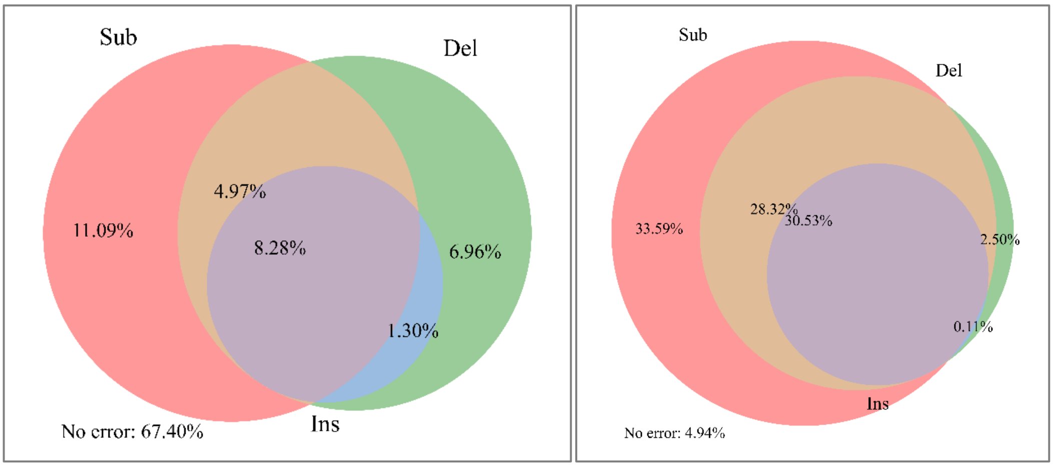

Figure S6. Read distribution by error combination of R2 reads (Left: PF read, Right: NPF read)

S4.7. Average error rate per base position

To analyze the positional tendencies of errors, we calculated the error rate at each nucleotide position by dividing the number of errors at that position by the total number of nucleotides. The total number of nucleotides was obtained by multiplying the oligo sequence length (152 nt) by the number of reads. For the position, we used the 'spos' value from the return of the editops function which was employed for mapping as described in Section S4.1. The range of 'spos' spans from 0 to (number of bases - 1). As noted in Section S4.1, since R1 and R2 reads are 150 nt and compared against 152 nt oligo sequences, positions up to 151 can appear in the error annotations for these reads.

We provided base-wise error rates not only for R1 and R2 reads but also for merged reads. As known in the previous sequencing studies, errors tend to occur more frequently near the ends of reads [R21-22], with the last base showing particularly high instability. In the case of all merged reads as well as R1 and R2 reads, we observed that deletion errors are concentrated around the middle positions. This is due to the accumulation of cases where consecutive deletions occur from the last valid base position to the end of the oligo sequence, caused by adapter trimming (for R1 and R2 reads) and short overlaps (in merged reads).

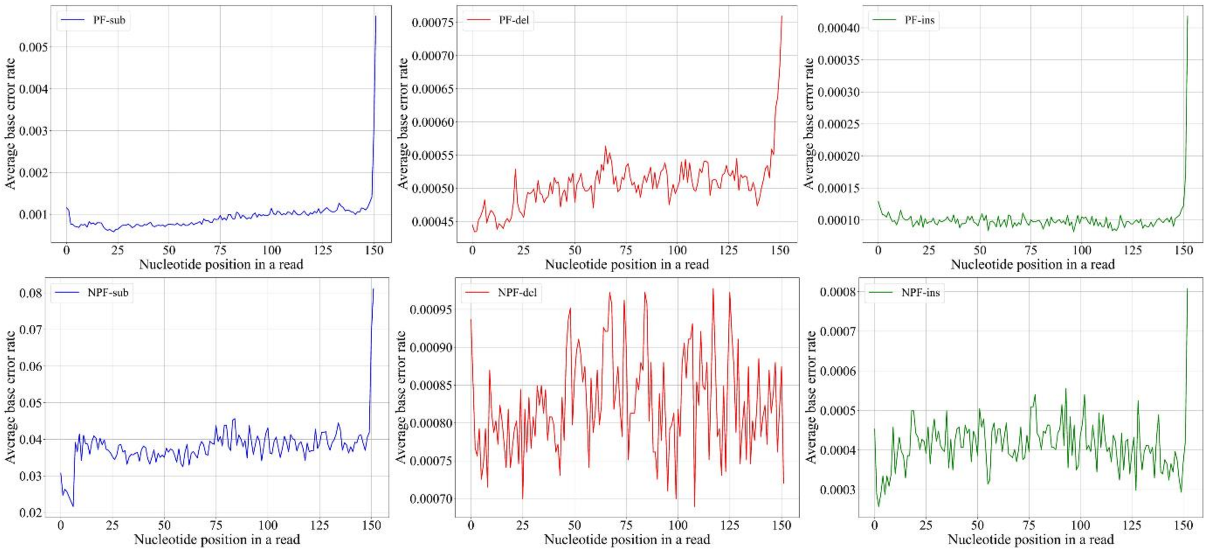

Figure S7. Error rate per nucleotide position of target-length reads (Top: PF read, Bottom: NPF read)

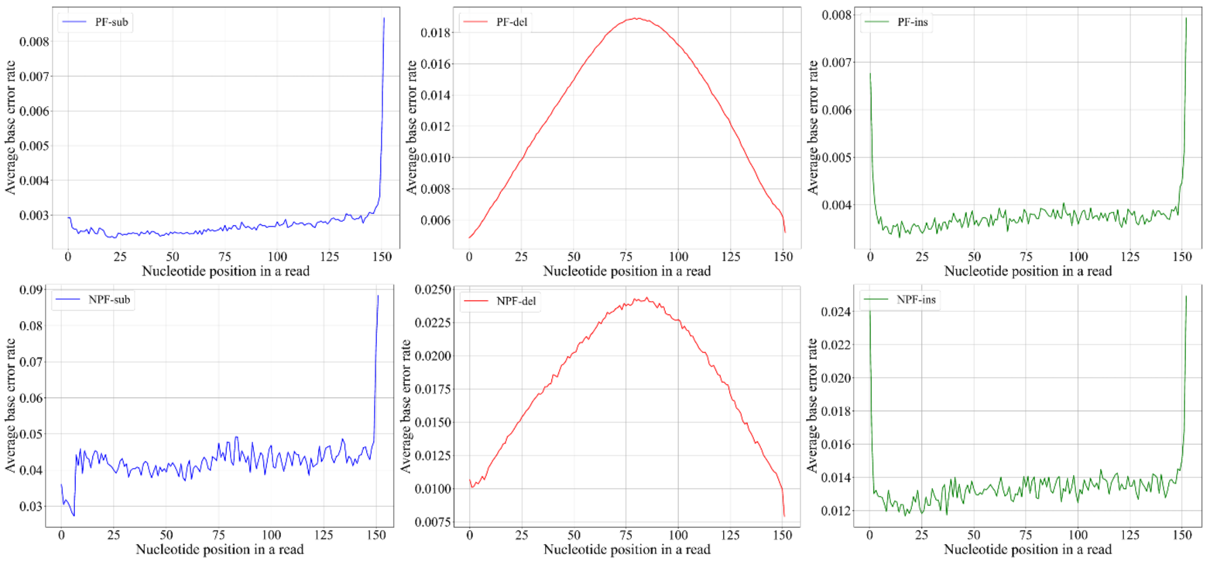

Figure S8. Error rate per nucleotide position of all merged reads (Top: PF read, Bottom: NPF read)

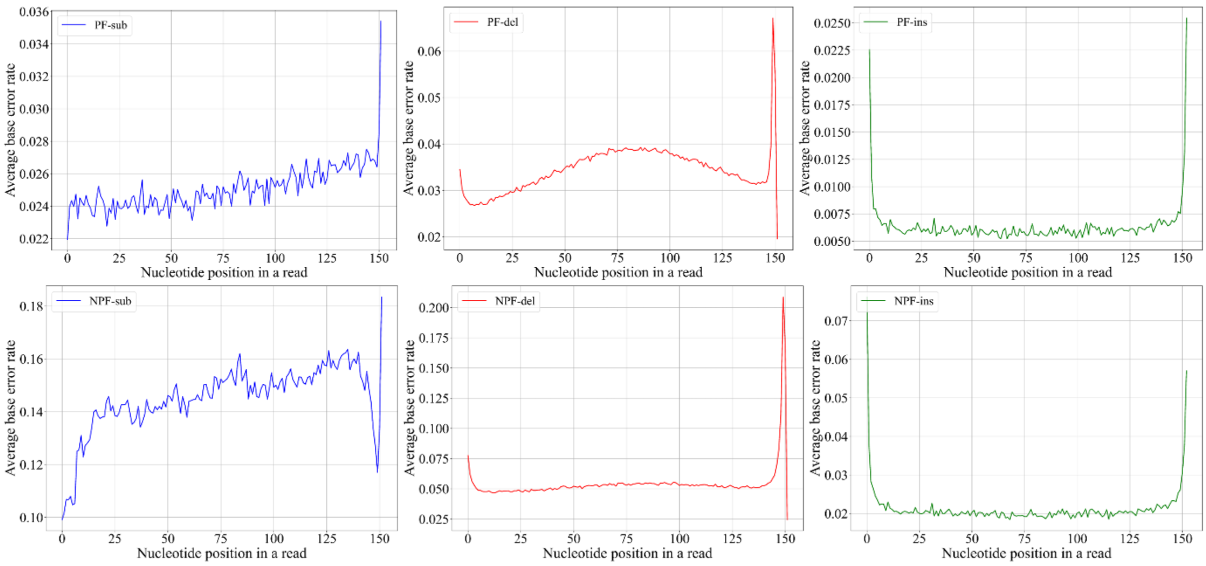

Figure S9. Error rate per nucleotide position of R1 reads (Top: PF read, Bottom: NPF read)

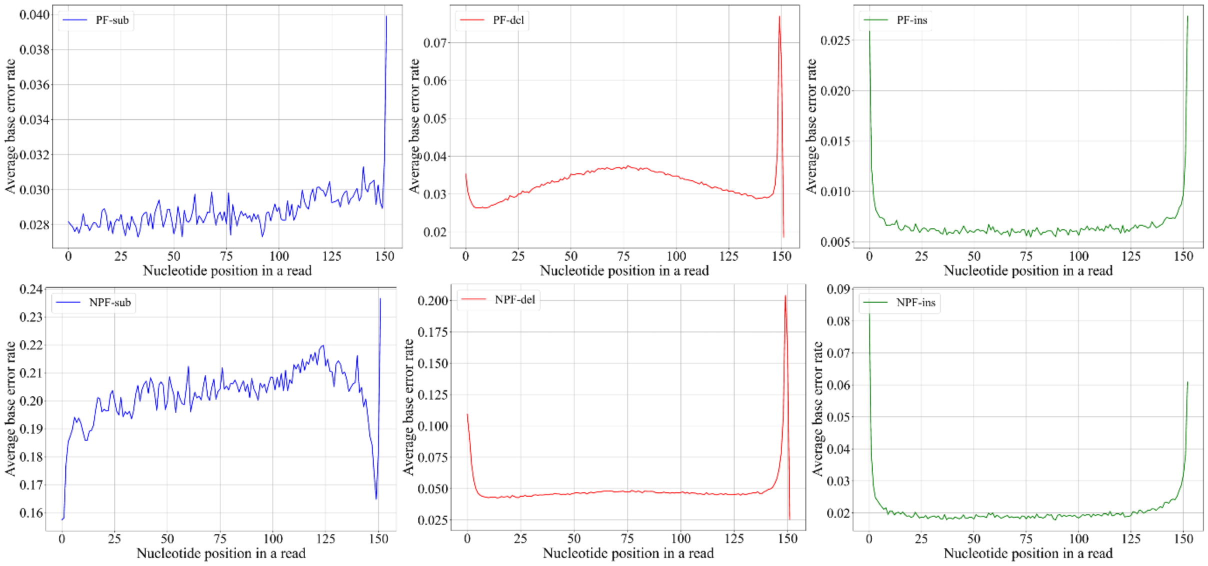

Figure S10. Error rate per nucleotide position of R2 reads (Top: PF read, Bottom: NPF read)

S4.8. Error analysis with other works

In our environment, the error rates of target-length PF reads are 0.0009 (Substitution), 0.0005 (Deletion), and 0.0001 (Insertion). In NPF reads, the error rates are 0.0384 (Substitution), 0.0008 (Deletion), and 0.0004 (Insertion). (See Section S4.2. for details.) Our indel error rate is 0.0006 for PF reads and 0.0012 for NPF reads. Compared to the indel error rate of 0.0218 reported in [R10], these values are 36.33 times and 18.17 times lower, respectively.

Table S26. Analysis of base error rates in studies using inner marker codes

Year	Reference	Substitution	Deletion	Insertion	Total	In-vitro experiment
2023	R1	3.00%	0.65%	0.65%	4.30%	X
2024	R2	0.80%	2.00%	2.00%	4.80%	X
2019	R3	0.30%	0.40%	0.40%	1.10%	X
2020	R4	1.00%	2.10%	2.10%	5.20%	X
2023	R5	0.00%	25%	25%	50.00%	X
2021	R6	0.45%	0.15%	0.05%	0.65%	X
2022	R7	1.00%	0.50%	0.50%	2.00%	X
2023	R8	2.20%	2.00%	1.70%	5.90%	X
2020	R9	X	1.00%	X	≥1.00%	X
2023	R10	4.28%	1.48%	0.70%	6.46%	O*
2021	R11	2.20%	2.00%	1.70%	5.90%	O
2021	R12	3.59%	4.07%	3.13%	10.79%	O
2019	R13	0.40%	0.85%	0.05%	1.30%	O
2023	R14	7.60%	10.20%	8.80%	26.60%	O

In cases where in-vitro experiments were not conducted, the highest indel error rate was reported.

* In-vitro experiments were conducted but the error analysis is based on the results from MESA (a DNA storage simulator). We reported the error rate when their proposed method achieved the perfect data recovery.

References

[R1] Tong, Jian, Guojun Han, and Yi Sun. "An Improved Marker Code Scheme Based on Nucleotide Bases for DNA Data Storage." *Applied Sciences* 13.6 (2023).

[R2] G. Ma, X. Jiao, J. Mu, H. Han and Y. Yang, "Deep Learning-Based Detection for Marker Codes Over Insertion and Deletion Channels." in *IEEE Transactions on Communications*, vol. 72, no. 10, pp. 5945-5959, (2024).

[R3] T. Xue and C. M. Francis Lau, "Concatenated Synchronization Error Correcting Code with Designed Markers." *2019 IEEE 5th International Conference on Computer and Communications (ICCC)*, Chengdu, China, pp. 1371-1376, (2019).

[R4] H. Kaneko, "Interleaved Non-Binary LDPC Code for Synchronization Error Correction in DNA Storage." *2020 IEEE International Conference on Consumer Electronics - Taiwan (ICCE-Taiwan)*, Taoyuan, Taiwan, (2020).

[R5] I. Maarouf, G. Liva, E. Rosnes and A. G. i. Amat, "Finite Blocklength Performance Bound for the DNA Storage Channel." *2023 12th International Symposium on Topics in Coding (ISTC)*, Brest, France, (2023).

[R6] K. Cai, Y. M. Chee, R. Gabrys, H. M. Kiah and T. T. Nguyen, "Correcting a Single Indel/Edit for DNA-Based Data Storage: Linear-Time Encoders and Order-Optimality." in *IEEE Transactions on Information Theory*, vol. 67, no. 6, pp. 3438-3451, (2021).

[R7] Z. Yan, C. Liang and H. Wu, "A Segmented-Edit Error-Correcting Code With Re-Synchronization Function for DNA-Based Storage Systems." in *IEEE Transactions on Emerging Topics in Computing*, vol. 11, no. 3, pp. 605-618, (2023).

[R8] L. Welter, I. Maarouf, A. Lenz, A. Wachter-Zeh, E. Rosnes and A. G. I. Amat, "Index-Based Concatenated Codes for the Multi-Draw DNA Storage Channel." *2023 IEEE Information Theory Workshop (ITW)*, Saint-Malo, France, (2023).

[R9] M. Cheraghchi, R. Gabrys, O. Milenkovic and J. Ribeiro, "Coded Trace Reconstruction." in *IEEE Transactions on Information Theory*, vol. 66, no. 10, pp. 6084-6103, (2020).

[R10] Welzel, M., Schwarz, P.M., Löchel, H.F. *et al.* "DNA-Aeon provides flexible arithmetic coding for constraint adherence and error correction in DNA storage." *Nat Commun* 14, 628 (2023).

[R11] S. R. Srinivasavaradhan, S. Gopi, H. D. Pfister and S. Yekhanin, "Trellis BMA: Coded Trace Reconstruction on IDS Channels for DNA Storage." *2021 IEEE International Symposium on Information Theory (ISIT)*, Melbourne, Australia, pp. 2453-2458, (2021).

[R12] Weigang Chen, Mingzhe Han, Jianting Zhou, Qi Ge, Panpan Wang, Xinchen Zhang, Siyu Zhu, Lifu Song, Yingjin Yuan, "An artificial chromosome for data storage." *National Science Review*, Volume 8, Issue 5 (2021).

[R13] S. Chandak *et al.*, "Improved read/write cost tradeoff in DNA-based data storage using LDPC codes." *2019 57th Annual Allerton Conference on Communication, Control, and Computing (Allerton)*, Monticello, IL, USA, pp. 147-156, (2019).

[R14] Yu, M., Lim, D., Kim, J., & Song, Y. "Processing DNA storage through programmable assembly in a droplet-based fluidics system." *Advanced Science*, *10*(32), (2023).

[R15] Yaniv Erlich, Dina Zielinski , "DNA Fountain enables a robust and efficient storage architecture." *Science*, 355, 950-954 (2017).

[R16] Jiajie Zhang, Kassian Kobert, Tomáš Flouri, Alexandros Stamatakis, "PEAR: a fast and accurate Illumina Paired-End reAd mergeR." *Bioinformatics*, Volume 30, Issue 5, Pages 614–620, (2014).
[R17] Shifu Chen, Yanqing Zhou, Yaru Chen, Jia Gu, "fastp: an ultra-fast all-in-one FASTQ preprocessor.' *Bioinformatics*, Volume 34, Issue 17, Pages i884–i890, (2018).
[R18] Schirmer, M., D'Amore, R., Ijaz, U.Z. *et al.* "Illumina error profiles: resolving fine-scale variation in metagenomic sequencing data." *BMC Bioinformatics* 17, 125 (2016).
[R19] Gimpel, Andreas L., et al. "Challenges for error-correction coding in DNA data storage: photolithographic synthesis and DNA decay." *Digital Discovery* 3.12: 2497-2508, (2024).
[R20] Heckel, R., Mikutis, G. & Grass, R.N. "A Characterization of the DNA Data Storage Channel." *Sci Rep* 9, 9663 (2019).
[R21] Pfeiffer, F., Gröber, C., Blank, M. *et al.* "Systematic evaluation of error rates and causes in short samples in next-generation sequencing." *Sci Rep* 8, 10950 (2018).
[R22] Tan, G., Opitz, L., Schlapbach, R. *et al.* "Long fragments achieve lower base quality in Illumina paired-end sequencing.' *Sci Rep* 9, 2856 (2019).
